# ﻿A new genus of Orthopsyllidae Huys, 1990 (Copepoda, Harpacticoida) from the Pacific Ocean

**DOI:** 10.3897/zookeys.1266.171760

**Published:** 2026-01-09

**Authors:** Jinwook Back, Hyun Woo Bang

**Affiliations:** 1 Department of Taxonomy and Systematics, National Marine Biodiversity Institute of Korea, Seocheon, Chungchungnam-do, 33662, Republic of Korea Department of Taxonomy and Systematics, National Marine Biodiversity Institute of Korea Seocheon Republic of Korea; 2 Division of Biological Industry, Mokwon University, Seo-gu, Daejeon, 35349, Republic of Korea Mokwon University Daejeon Republic of Korea

**Keywords:** Crustacea, DNA barcode, new harpacticoids, taxonomy

## Abstract

This study reports the discovery of four new benthic harpacticoids from Korean waters in the Pacific Ocean. The harpacticoids were collected using SCUBA or a grab sampler and sorted using a stereomicroscope for both molecular and morphological analysis. The study employed standard DNA sequencing methods (COI and 18S rRNA) and detailed morphological characterization through microscopy and scanning electron microscopy. As a result, three new species belonging to the family Orthopsyllidae and classified as a new genus were discovered. Although the new genus lacks brush setae on leg 1, a diagnostic feature of Orthopsyllidae, it shares similarities with other orthopsyllids in characters such as the large thorn-like process on the antennule, leg 5, and sexual dimorphism. This finding necessitates an expanded diagnosis of Orthopsyllidae to reflect the characters of the new genus based on the three new species. In addition, a new species belonging to the genus *Orthopsyllus* was discovered. This study is the first to describe a new species of *Orthopsyllus* from Korea, where the genus has not been previously reported at the species level. This study provides a key for classifying genera and species within the Orthopsyllidae family.

## ﻿Introduction

**Figure 1.**
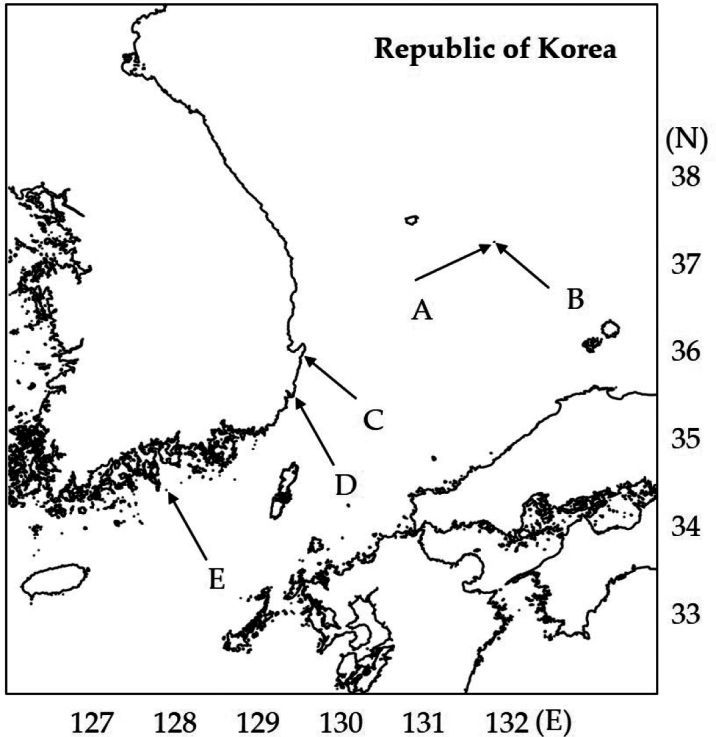
Map of the sampling sites and the dates. **A.** Near the western Dokdo island (37°14'22.69"N, 131°51'53.09"E, 2 November 2017); **B.** Near western Dokdo island (37°14'23.23"N, 131°52'20.06"E, 16 May. 2017); **C.** Near the Guryongpo port (35°55'30.6"N, 129°32'52.3"E, April 11, 2022); **D.** Near the Ilsan beach (35°29'27.67"N, 129°26'34.48"E, 26 April 2018); **E.** Near the Jakdo island (34°24'58.9"N, 127°54'20.4"E, 3 June 2020).

During research on the diversity of benthic harpacticoids, a new genus within Orthopsyllidae Huys, 1990 was found in Korean waters in the Pacific Ocean. The four new species were discovered in waters with an annual temperature range exceeding 7 °C (Mitchell et al. 2005) and were collected from sand or broken rock at a depth of approximately 10 m (Fig. 1). Two hundred and seventeen species of marine harpacticoids have been discovered near the Korean Peninsula (Marine Bio-Resource Information System [MBRIS] 2015). In this study, new harpacticoids that do not belong to the genus *Orthopsyllus* Brady & Robertson, 1873 were identified.

The superfamily Laophontoidea consists of six families: Normanellidae Lang, 1944; Laophontidae T. Scott, 1904; Adenopleurellidae Huys, 1990; Orthopsyllidae Huys, 1990; Cristacoxidae Huys, 1990; Laophontopsidae Huys & Willems, 1989, comprising 405 species (Walter and Boxshall 2025). The relationship between Laophontoidea and its morphological characters has been discussed in several studies (Huys 1990; Huys and Lee 1998; Gómez and Nazari 2021). Nevertheless, the classification of species belonging to Laophontoidea requires careful consideration due to the presence of subspecies and morphological variations among them (Boxshall and Halsey 2004; Wells 2007).

Huys (1990) moved the genus *Orthopsyllus* Brady & Robertson, 1873 from Canthocamptidae Brady, 1880 to a new family. Although no formal diagnosis was provided, he listed seven apomorphic characters of Orthopsyllidae. Huys and Lee (1998) also described 30 characters that analyzed the taxonomic relationship among six laophontoidan families, which are useful for identifying the features of the family Orthopsyllidae. Orthopsyllidae can be classified by features such as: 1) 4-segmented antennule; 2) a hook-like process in the second segment of the antennule; 3) brush setae in P1 endopod and exopod; 4) a strongly reduced proximal segment on the endopod of the second to fourth appendages; and 5) sexual dimorphism in the endopod of the second and third appendages in males (Huys 1990; Huys and Lee 1998). To date, the family comprises 15 species and three subspecies within a single genus (Walter and Boxshall 2025). Huys (1990) also noted that the structures of the mouth are important morphological characters in taxonomic studies but detailed descriptions have been omitted for many species. DNA sequences that could aid in species identification are currently unavailable for previously described species (National Center for Biotechnology Information [NCBI] 2025), making phylogenetic studies currently impossible at present. This study provides an expanded diagnostic key to the genera and species of the family Orthopsyllidae including a new genus and four new species.

## ﻿Materials and methods

### ﻿Sample collection

The sediment samples were collected using SCUBA or a grab sampler and fixed with > 99% ethanol (Fig. 1). Harpacticoids were sorted from samples using a Leica M80 stereomicroscope. The sorted specimens were kept refrigerated at –20 °C prior to DNA extraction.

### ﻿DNA extraction, sequencing, and analysis

The general processes for DNA extraction and polymerase chain reaction (PCR) were adopted from Lim et al. (2020). Both mitochondrial COI and 18S rRNA sequences were amplified from the DNA samples using an AccuPower HotStart PCR PreMix (Bioneer, Daejeon, South Korea). PCR products were sequenced in both directions using an ABI PRISM 3730XL analyzer (Macrogen Inc., Korea). Sequences were aligned using the Muscle algorithm integrated into Prime 2024.0.5 (Edgar 2004). Geneious Prime 2025.2.2 (Biomatters, Auckland, New Zealand) was used to assemble the sequences (Kearse et al. 2012). The molecular phylogenetic tree was reconstructed based on concatenated mtCOI and 18S rRNA sequences obtained from the Genbank. The information of the sequences used was summarized in Table 1. The alignments of each gene set were constructed using MAFFT version 7.520 (Katoh and Standley 2013) with the L-INS-i algorithm. Phylogenetic relationships were inferred through Maximum Likelihood analysis under the Tamura-Nei (TN93) with a Gamma distribution (+G) and evolutionarily invariable (+I) (Nei and Kumar 2000), as determined by the model selection in MEGA X (Kumar et al. 2018). Node support values were assessed using 1,000 bootstrap replicates. The tree was rooted with *Scottolana daecheonensis* Bang, Moon & Back, 2022.

### ﻿Morphological characterization

After processing for molecular analysis, each specimen was dissected onto several slides using lactophenol as the mounting medium and observed using a Leica DM2500 microscope equipped with a drawing tube. Specimens for scanning electron microscopy (SEM) were dehydrated using a t-BuOH freeze-dryer (VFD-21S; Vacuum Device, Ibaraki, Japan). They were mounted on stubs and coated with gold-palladium. SEM observations were performed using an SEM (SU3500, Hitachi for Figs 9, 10, 17; Zeiss Sigma 500 VP, Carl Zeiss for Fig. 36). The descriptive terminology was adopted from Huys et al. (1996). Abbreviations used in the systematics, description, and figure and discussion are: **A1**: antennule; **A2**: antenna; **ae**: aesthetasc; **Md**: mandible; **Mxl**: maxillule; **Mxa**: maxilla; **Mxp**: maxilliped; **exp**: exopod; exp–1(2,3): proximal (middle, distal) exopod; **enp**: endopod; **enp–1(2,3)**: proximal (middle, distal) endopod; **P1–P6**: first to sixth thoracopod; **benp**: baseoendopod; **seg**: segment; **seg–1(–5)**: first (to fifth) segment. The specimens were deposited in the Marine Biodiversity Institute of Korea (**MABIK**; Seocheon, Republic of Korea). Collection numbers are provided in parentheses alongside each specimen.

## ﻿Systematics

### ﻿Class Copepoda Milne Edwards, 1840

**Order Harpacticoida Dana, 1846**

**Family Orthopsyllidae Huys, 1990**

#### ﻿Genus Intercristacoxa gen. nov.

Taxon classificationAnimaliaHarpacticoidaOrthopsyllidae

F3F16D08-2216-5494-842E-09C973E68896

https://zoobank.org/FDE0568E-A8C7-4AD5-BEAF-1313116BDCF9

##### Diagnosis.

Orthopsyllidae. Female. Body nearly cylindrical. Rostrum well developed and defined at base. Genital double-somite separated dorsally and laterally, but fused ventrally. A1 four-segmented, with one developed thorn-like process on seg–1; apical ae absent. A2 consisting of coxa, allobasis without abexopodal seta, one-segmented enp, and one-segmented exp. Mandibular palp with one-segment enp. Enp of Mxl incorporated into basis with three setae; exp one-segmented with two setae. Syncoxa of Mxa with two endites, proximal endite with one seta; basis drawn out into claw; enp incorporated into the basis with three setae. Mxp subchelate, three-segmented; basis slender, elongated; enp represented by an acutely recurved claw. The P1 coxa with one or two serrate crests; exp one–segmented, with two long setae, without spine; enp two-segmented; enp–1 elongated and without inner seta; enp–2 rectangular, with one curved claw and one cylindrical seta. P3–P4 exp each with two spines. P5 benp well developed; exp defined at the base. Caudal rami seven setae, with seta V being bi-articulated. Sexual dimorphism in body size, A1, P3 enp, P4 enp, P5, P6, and genital segmentation.

##### Type species.

*Intercristacoxa orientalis* gen. et sp. nov. by designation.

##### Etymology.

The generic name is a combination of the Latin *inter*-, meaning between, and *crista coxa*, which refers to the coxal crest of the first leg, and is treated as feminine.

#### ﻿ Intercristacoxa orientalis sp. nov.

Taxon classificationAnimaliaHarpacticoidaOrthopsyllidae

8C2948A9-7948-5805-A7DE-9B6F0DAA99F1

https://zoobank.org/46D6D801-83F1-47D5-BEBB-177B72C8EFFA

Figs 2, 3, 4, 5, 6, 7, 8, 10

##### Type locality.

Subtidal, site A (37°14'22.69"N, 131°51'53.09"E, Fig. 1), depth 7–10 m, sand between cracks in stone, 16 November 2017.

##### Type material.

***Holotype***: • 1♀ (MABIK CR00258585), dissected on nine slides. ***Paratypes***: • 1♂ (MABIK CR00258583), dissected on nine slides; • 1♀ (MABIK CR00258584), dissected on eight slides for confirmation. Three specimens (1♀, 2♂♂) dried, mounted on stub, and coated with gold for SEM.

##### GenBank accession number.

Mitochondrial cytochrome c oxidase subunit I genes (PX210751, PX210752, PX485055–PX485058) and 18S ribonucleic acid genes (PX218728 and PX218729).

##### Description.

**Figure 2.**
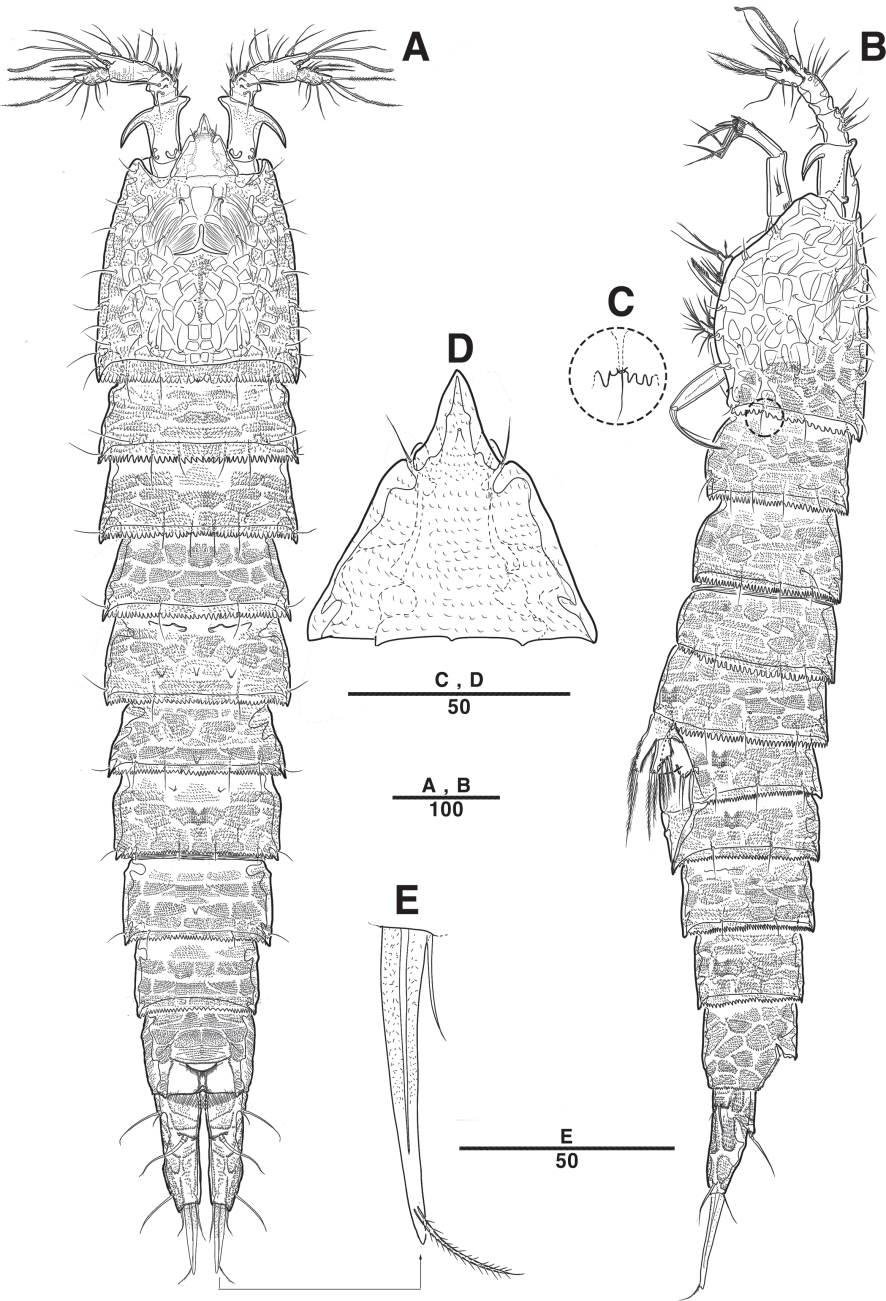
*Intercristacoxa orientalis* sp. nov., ♀, MABIK CR00258585. **A.** Habitus, dorsal; **B.** Habitus, lateral; **C.** Distal hyaline frill of somites; **D.** Rostrum; **E.** Caudal seta IV and V. Scale bars in μm.

**Figure 3.**
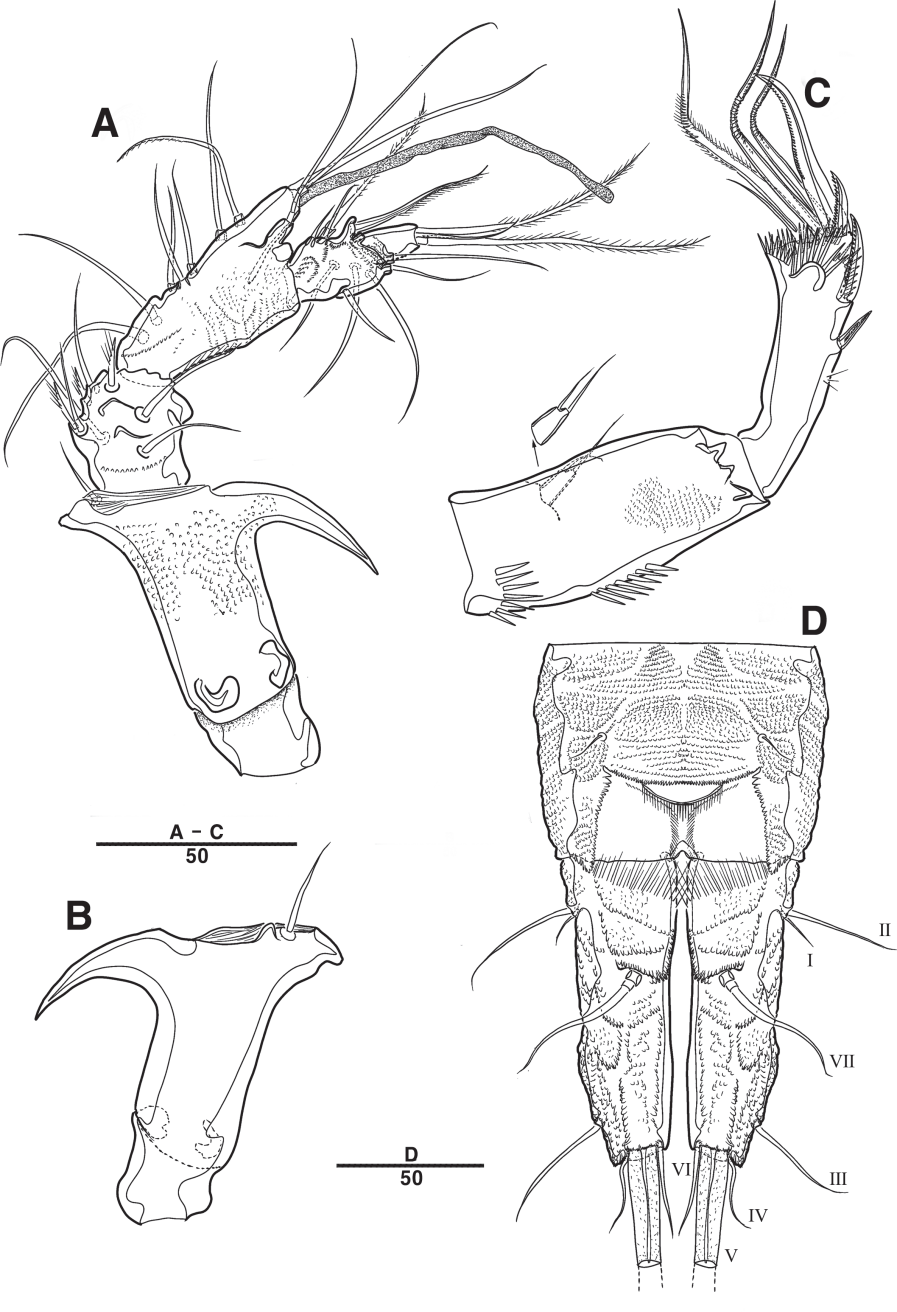
*Intercristacoxa orientalis* sp. nov., ♀, MABIK CR00258585. **A.**A1; **B.**A1seg–1; **C.**A2; **D.** Caudal rami, dorsal. Scale bars in μm.

Female. Total body (Fig. 2A, B) nearly cylindrical, with minute sensilla dorsally, covered by pattern of sensilla and pores as illustrated, length from anterior margin of rostrum to posterior margin of caudal rami 1,000 μm, maximum width 180 μm measured at midway of cephalothorax; hyaline frill bumpy (Fig. 2C); rostrum (Fig. 2D) well developed, defined at base, triangular, with distal third tapering, with two sensilla; cephalothorax wider than free somites (Fig. 2A), pleural areas of cephalic shield narrow and posterolateral angles rounded; second and third urosomites (genital double somite) separated dorsally and laterally (Fig. 2A, B), fused ventrally but discontinuous internal chitinous rib indicating original segmentation (Fig. 5A), genital apparatus located mid-ventrally at anterior half of genital double somite, genital pores covered by P6 (Fig. 5E); anal operculum not developed (Fig. 3D).

Caudal rami (Figs 3D, 5B), parallel, ~ 2.6 × longer than maximum width, surface armed with minute spinules (also see Fig. 10C in male); each ramus with seven setae; seta IV fused at base to seta V; seta V bi-articulated, consists of spine-like seta at top and small pinnate seta attached at end (Fig. 2E); seta VI bare at inner distal corner, seta VII tri-articulate at base, positioned dorsally on upper one–third of caudal rami.

A1 (Figs 3A, B, 9A); slender, four-segmented; seg–1 large, with one thorn-like process at distal outer corner; seg–3 with sub-cylindrical pedestal with ae fused at base to one long naked seta; seg–4 representing two fused segments but middle horizontal ridge indicating original segmentation; armature formula: 1–[1], 2–[8], 3–[9 + (1 + ae)], 4–[13], apical ae not observed.

A2 (Fig. 3C): allobasis with two rows of spinules, without abexopodal setae; exp one-segmented, with two bare setae; and enp with four spines, three geniculate setae, and one bare seta.

**Figure 4.**
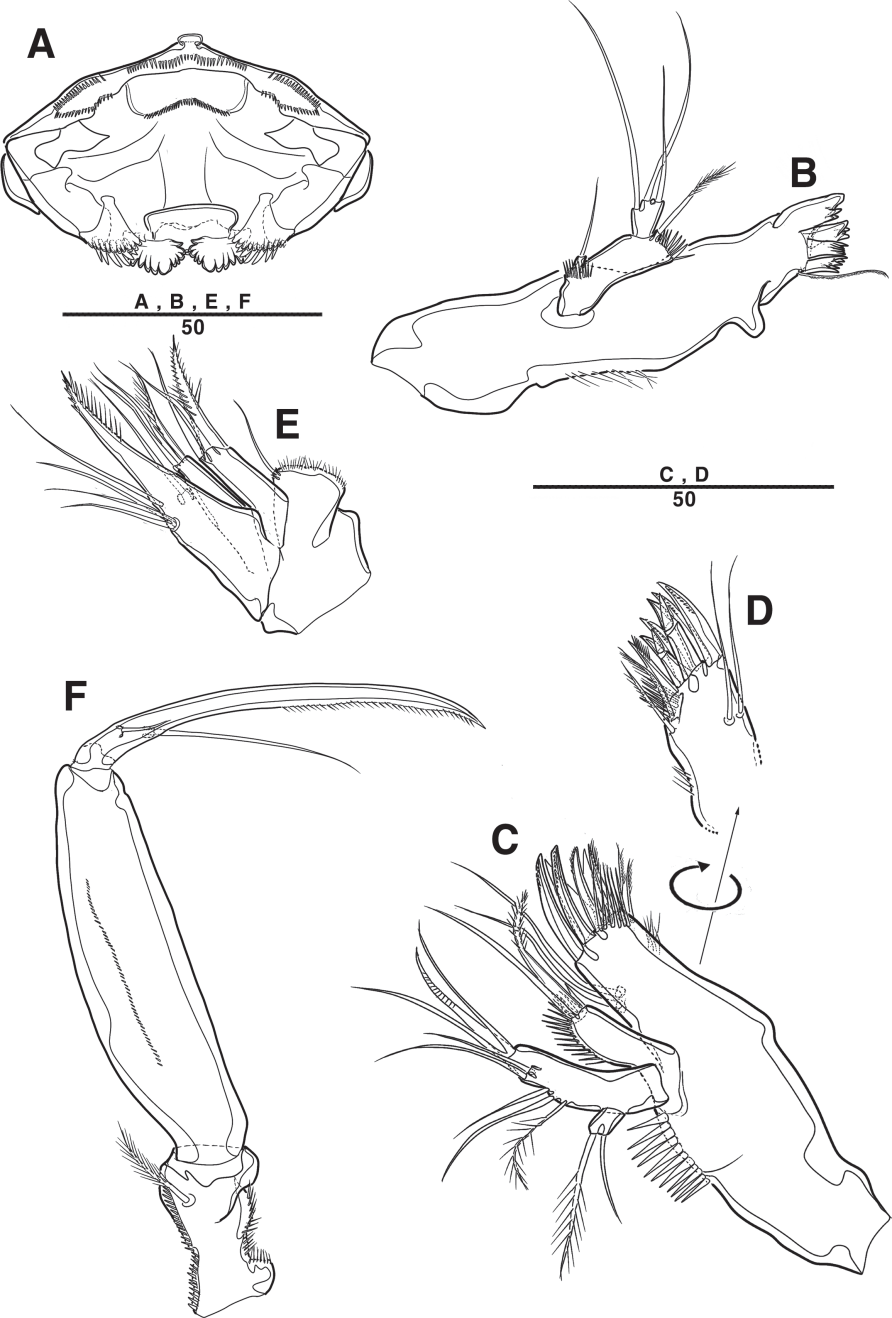
*Intercristacoxa orientalis* sp. nov., ♀, MABIK CR00258585. **A.** Labrum; **B.**Md; **C.**Mxl; **D.** Arthrite of Mxl, anterior; **E.**Mxa; **F.**Mxp. Scale bars in μm.

Labrum (Figs 4A, 9B): dense pattern of blunt processes in middle and distal patches of overlapping scales.

**Figure 5.**
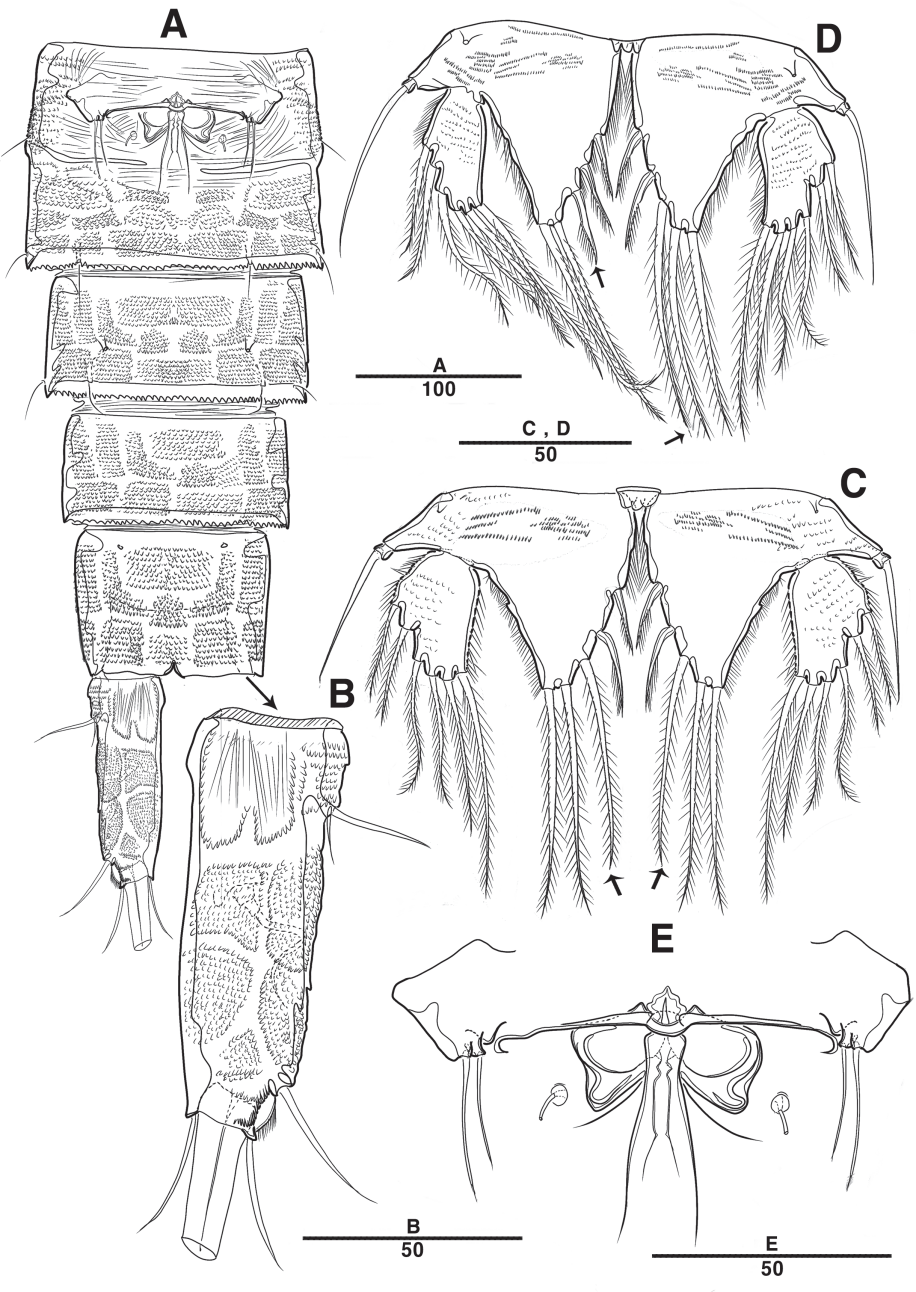
*Intercristacoxa orientalis* sp. nov., ♀, MABIK CR00258585. **A.** Urosome, ventral; **B.** Caudal ramus, ventral; **C.** P5; **D.** Abnormality in P5 (MABIK CR00258584); **E.** P6 and genital field. Scale bars in μm.

Md (Fig. 4B): gnathobase small, with one uni-pinnate seta laterally and several multi-cuspidate teeth; palp basis with one bare seta on inner margin; enp one-segmented, with three bare setae; exp incorporated into basis, represented by small protrusion with one bare seta.

Mxl (Fig. 4C, D): praecoxa trapezoidal; arthrite well developed with two juxtaposed bare setae on anterior surface, two pinnate setae laterally, and six elements along distal margin, ornamented with a row of setules along inner margin; coxa with one bare and one pinnate setae; basis with three distal setae fused at base and two proximal setae; enp incorporated in basis, represented by small protrusion, with one pinnate and two bare setae; exp one-segmented, with one bare and one pinnate setae.

Mxa (Fig. 4E): syncoxa with three endites; proximal endite vestigial, represented by one seta; second endite with one strong spine and two bare setae; distal endite with three setae; allobasis produced into strong curved claw, accessory armature consisting of two setae; and enp represented by three bare setae fused at base.

Mxp (Figs 4F, 9C): three-segmented; syncoxa with one pinnate seta; basis slender, elongate, bare; enp represented by an acutely recurved claw with two accessory setae.

**Figure 6.**
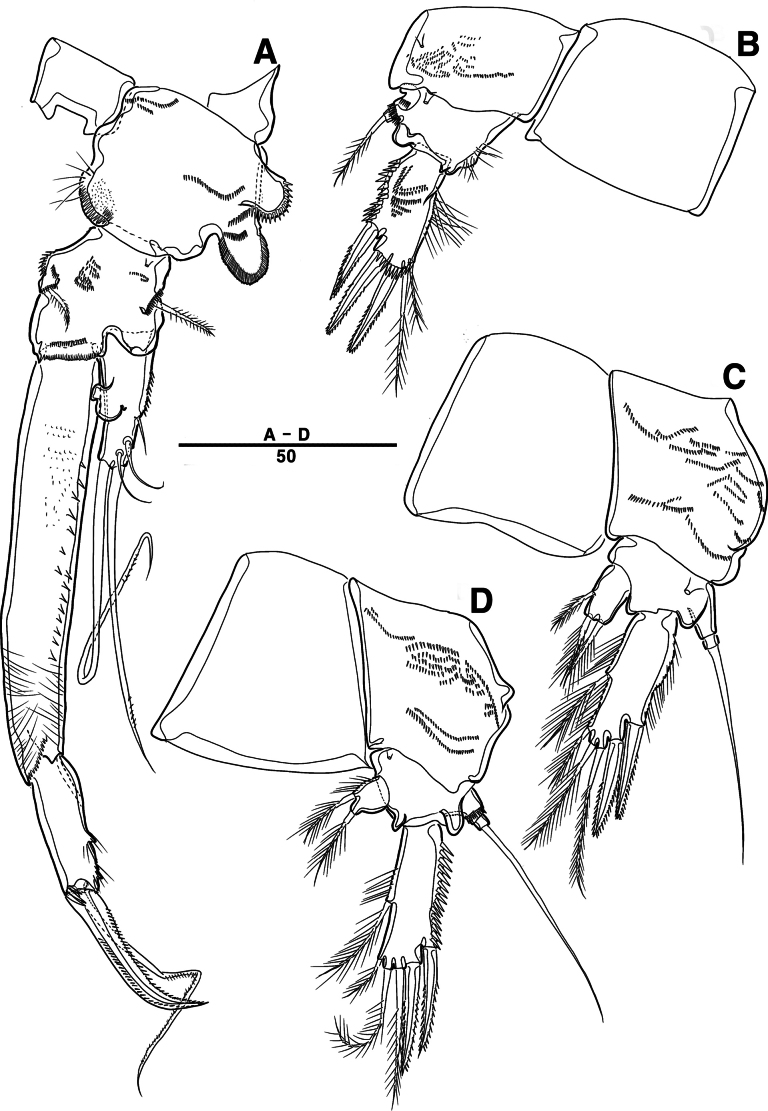
*Intercristacoxa orientalis* sp. nov., ♀, MABIK CR00258585. **A.** P1; **B.** P2; **C.** P3; **D.** P4. Scale bars in μm.

P1 (Figs 6A, 9C–E): coxa with two serrate crests (Fig. 9C, white arrow); basis with one outer and one inner pinnate setae, ornamented with small frills on base of enp (Fig. 9D); exp one-segmented, with three outer and two apical setae, ornamented with two protuberances (Fig. 9D, white arrow); enp two-segmented, prehensile; enp–1, elongate, ~ 5.2 × as long as wide, bare; enp–2, rectangular, with one curved claw and one seta (Fig. 9E).

P2 (Fig. 6B): coxa bare; basis with one outer pinnate seta; exp one-segmented, with three spines and two pinnate setae, ornamented with a tuft of setules laterally; enp absent.

P3 (Fig. 6C): coxa bare, basis with one outer bare seta; exp one-segmented, with two outer spines and four pinnate setae; enp one-segmented, with three pinnate setae.

P4 (Figs 6D, 9E): coxa bare; basis with one outer bare seta; exp one-segmented, with two outer spines and four pinnate setae; enp one-segmented, with two pinnate setae.

Armature formulae as follows:

**Table:** 

	Exp	Enp
P2	113	–
P3	222	120
P4	222	110

P5 (Figs 5C, D, 9F): basal part with one outer bare seta; endopodal lobes not fused medially, connected by intercoxal sclerite, with five pinnate setae; length of middle seta abnormal in paratype (Fig. 5D, black arrow); exp defined at base, with six pinnate setae.

P6 (Fig. 5A, E): P6 vestigial represented by a small bump with two bare setae.

**Figure 7.**
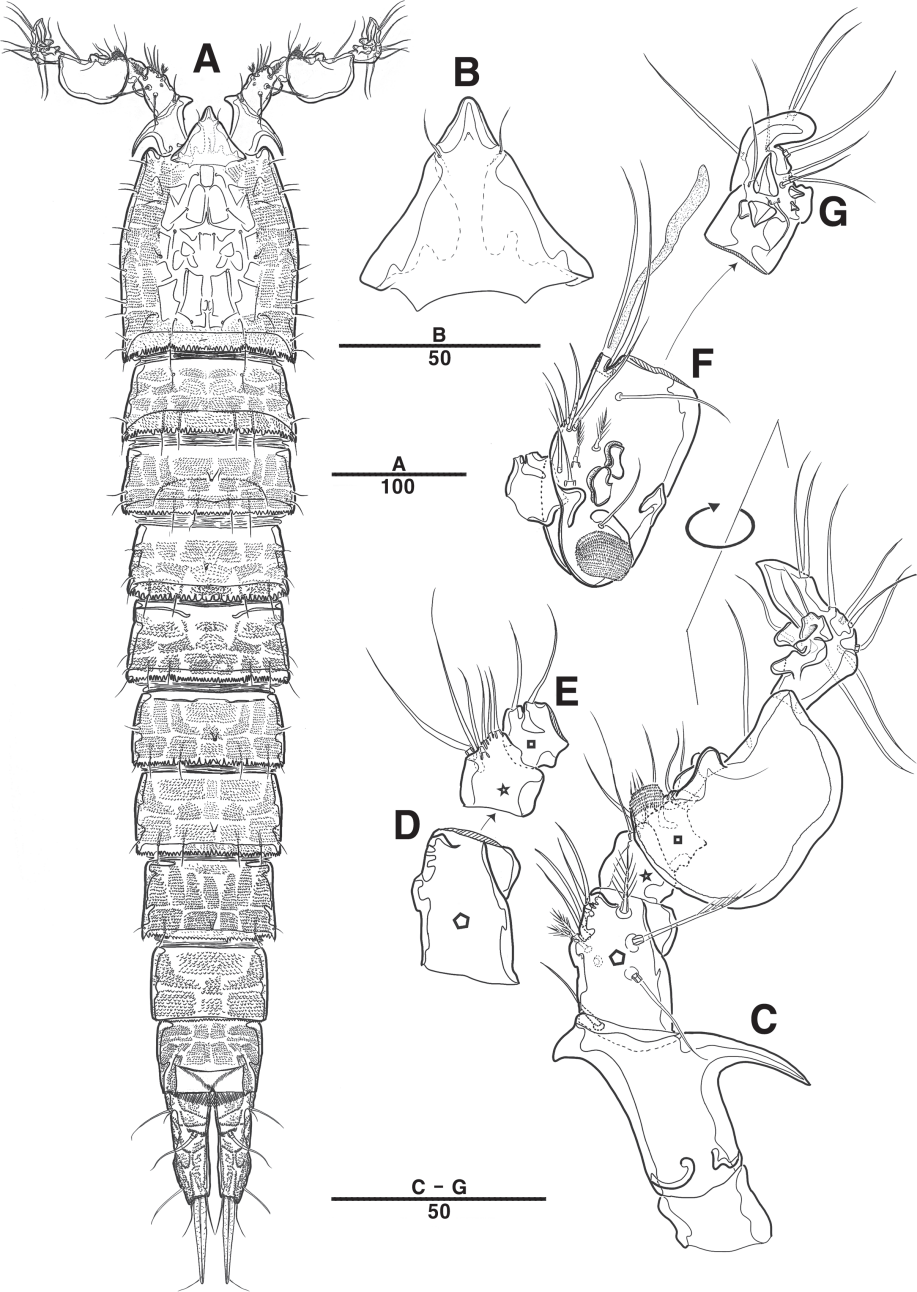
*Intercristacoxa orientalis* sp. nov., ♂, MABIK CR00258583. **A.** Habitus, dorsal; **B.** Rostrum; **C.**A1; **D.**A1seg–2; **E.**A1seg–3 and –4; **F.**A1seg–5; **G.**A1seg–6. Scale bars in μm.

Male. Total body length (Figs 7A, 10A) from anterior margin of rostrum to posterior margin of caudal rami 810 μm, maximum width 133 μm measured at midway of cephalothorax.

General body shape, ornamentation, and sensilla patterns as in female, except for genital double somite; sexual dimorphism in A1, P3, P4, P5, and P6.

A1 (Figs 7C–G, 10B), six segmented, robust, chirocer; seg–1 with one thorn-like process at distal outer corner; seg–5 swollen, with pad-like process near anterior margin; three processes in middle; ae fused at base to one bare seta, armature formula: 1–[1], 2–[9], 3–[6], 4–[2], 5–[11 + (1 + ae)], 6–[11], apical ae not observed.

Rostrum (Fig. 7B): apically blunter than that of female.

Setal formula and general shape of P1 and P2 similar to those of female.

**Figure 8.**
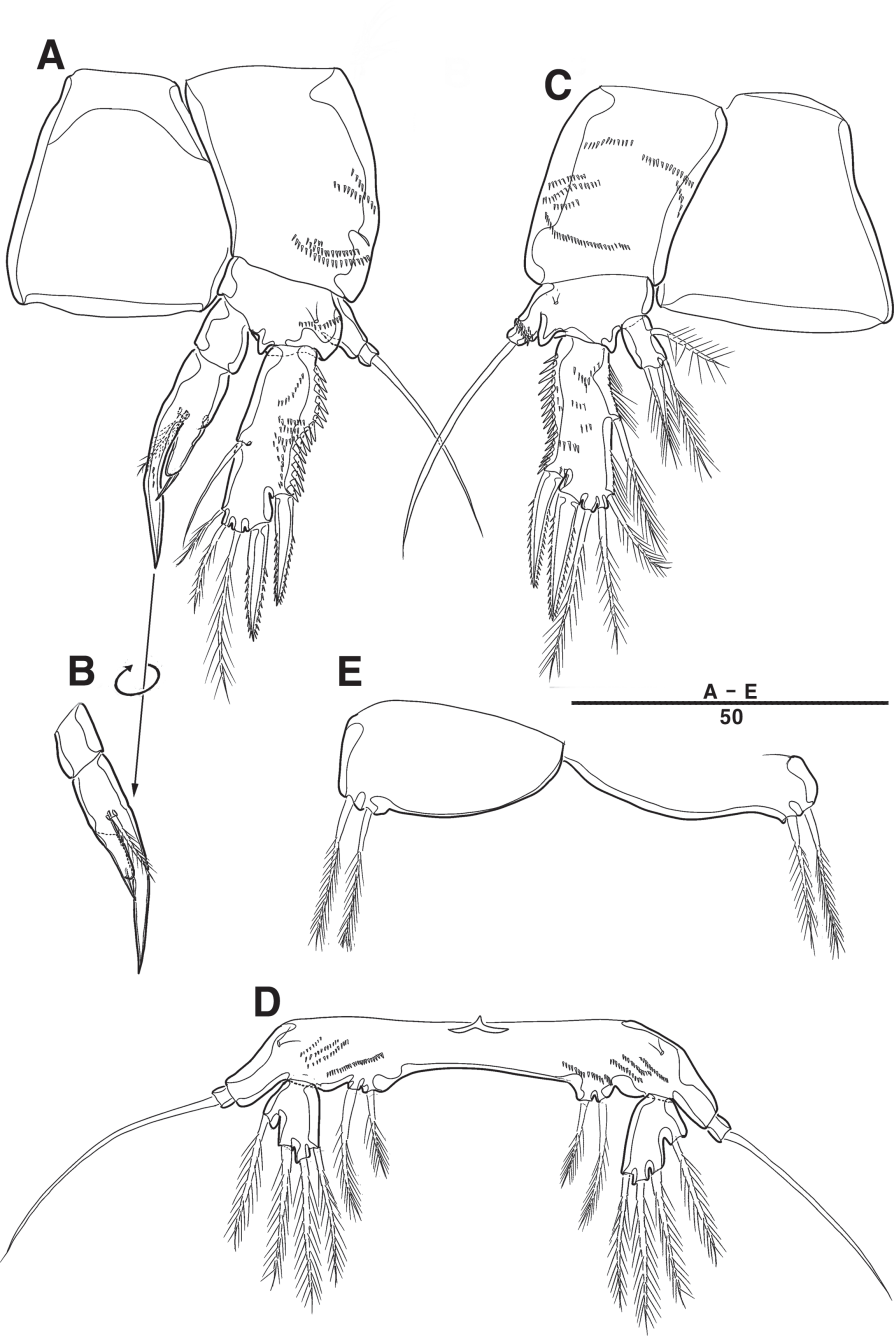
*Intercristacoxa orientalis* sp. nov., ♂, MABIK CR00258583. **A.** P3; **B.** Enp of P3; **C.** P4; **D.** P5; **E.** P6. Scale bars in μm.

P3 (Figs 8A, B, 10D): coxa bare; basis with one outer bare seta; exp one-segmented, with two spines and four setae; enp two-segmented; enp–1 small, bare; enp–2 elongated, armed with spinous apophysis, one lateral pinnate, and one apical minute setae.

**Figure 9.**
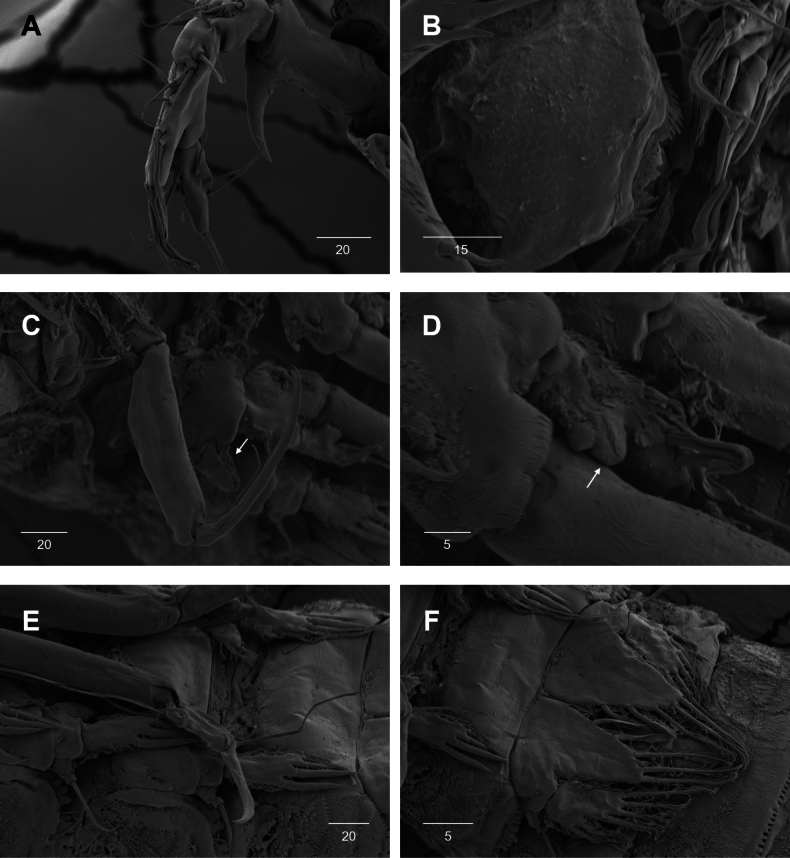
*Intercristacoxa orientalis* sp. nov., ♀, SEM. **A.**A1; **B.** Labrum; **C.**Mxp and P1 coxa; **D.** P1 enp; **E.** P1 exp and P3; **F.** P4 and P5. Scale bars in μm.

**Figure 10.**
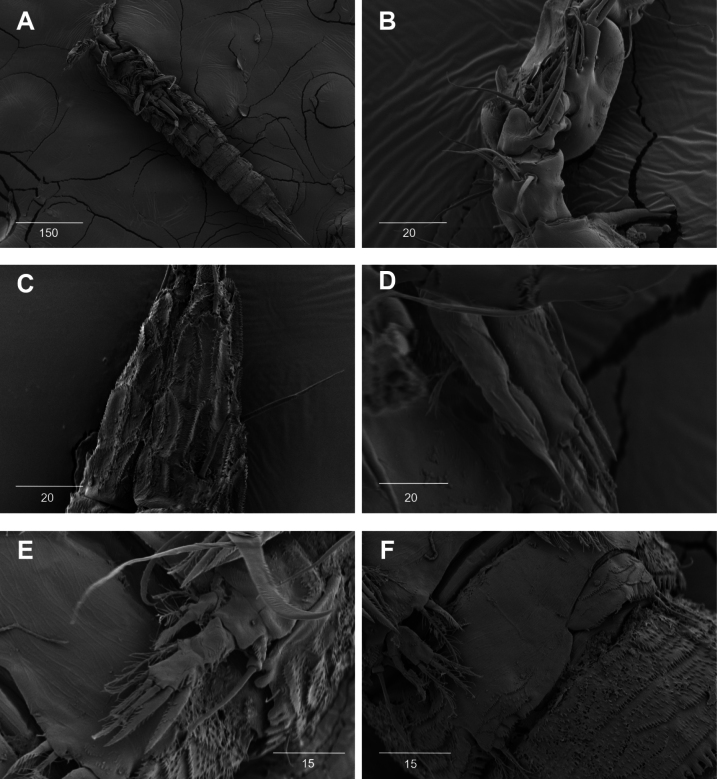
*Intercristacoxa orientalis* sp. nov., ♂, SEM. **A.** Habitus, ventral; **B.**A1seg–5; **C.** Caudal rami, lateral; **D.** P3; **E.** P4; **F.** P5 and P6. Scale bars in μm.

P4 (Figs 8C, 10E): coxa bare; basis with one outer bare seta; exp one-segmented, with two spines and four setae; enp one-segmented, with one apical seta (stouter than female) and two lateral pinnate setae.

P5 (Figs 8D, 10F): benp presented by a small protrusion with two pinnate setae; exp defined at base, with four pinnate setae.

P6 (Figs 8E, 10F): asymmetrical, with functional right member articulating at base and closing off genital aperture, and left member fused at base to genital somite; P6 with two pinnate setae.

##### Etymology.

The specific name is derived from the Latin adjective *orientalis*, meaning eastern, referring to the type locality in East Asia.

#### ﻿ Intercristacoxa koreana sp. nov.

Taxon classificationAnimaliaHarpacticoidaOrthopsyllidae

562CF7AC-74FB-5CA9-97FE-A97E80ADA70B

https://zoobank.org/5046E8DD-C377-413A-B205-7D37DF6D9A28

**Figure 11.**
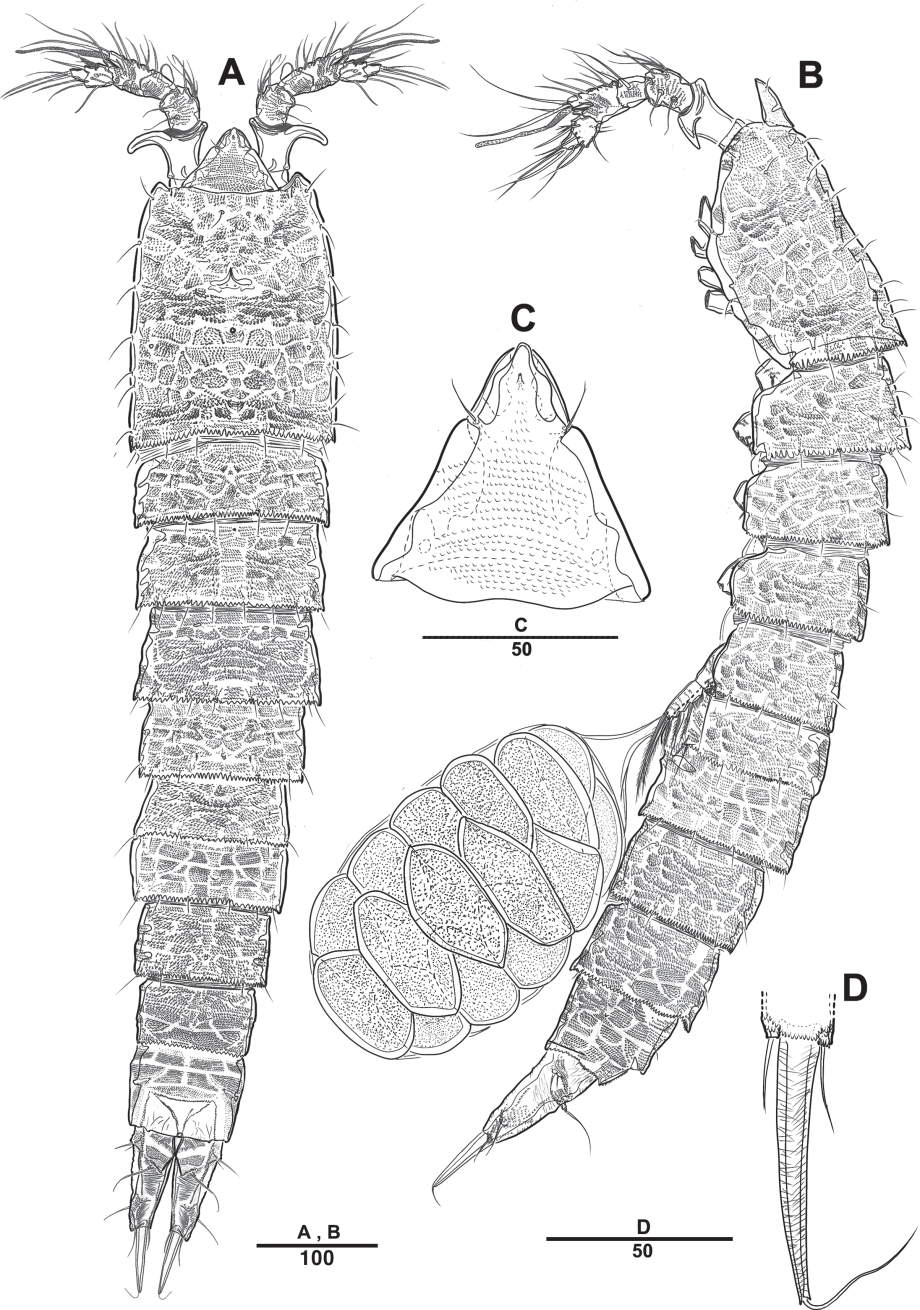
*Intercristacoxa koreana* sp. nov., ♀, MABIK CR00258582. **A.** Habitus, dorsal; **B.** Habitus, lateral; **C.** Rostrum; **D.** Caudal seta IV–VI. Scale bars in μm.

**Figure 12.**
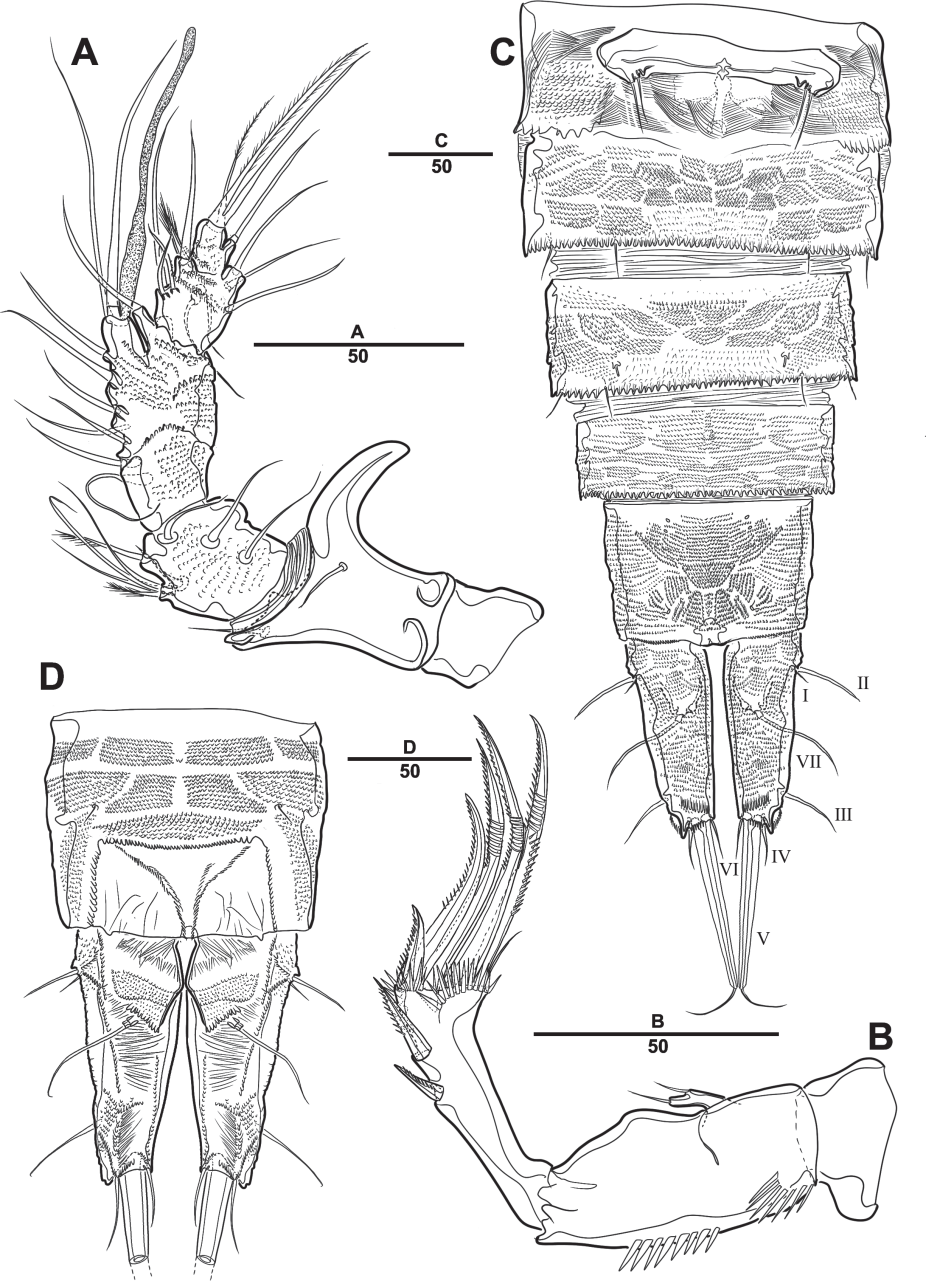
*Intercristacoxa koreana* sp. nov., ♀, MABIK CR00258582. **A.**A1; **B.**A2; **C.** Urosome, ventral; **D.** Anal somite and caudal rami. Scale bars in μm.

**Figure 13.**
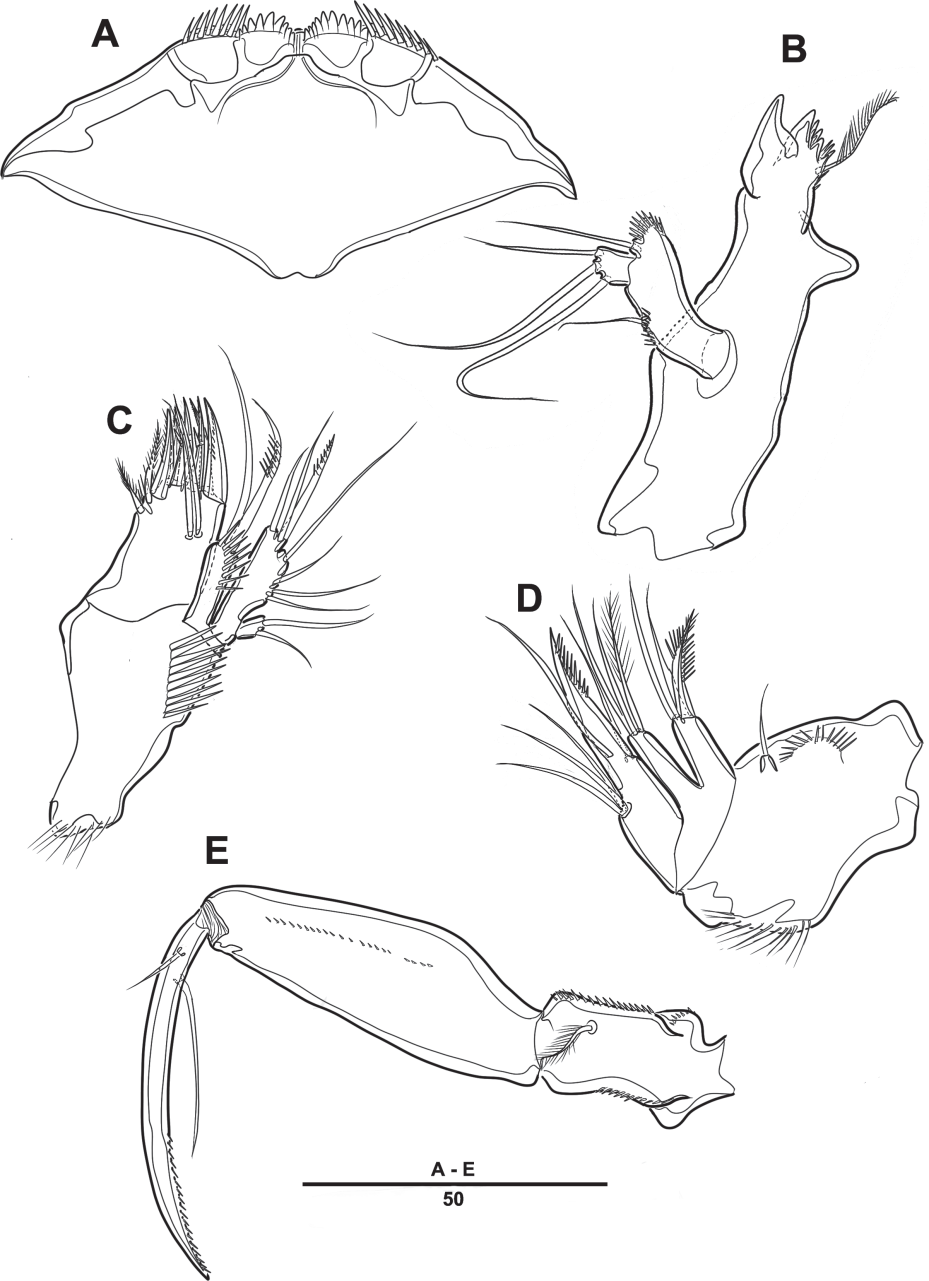
*Intercristacoxa koreana* sp. nov., ♀, MABIK CR00258582. **A.** Labrum; **B.**Md; **C.**Mxl; **D.**Mxa; **E.**Mxp. Scale bars in μm.

**Figure 14.**
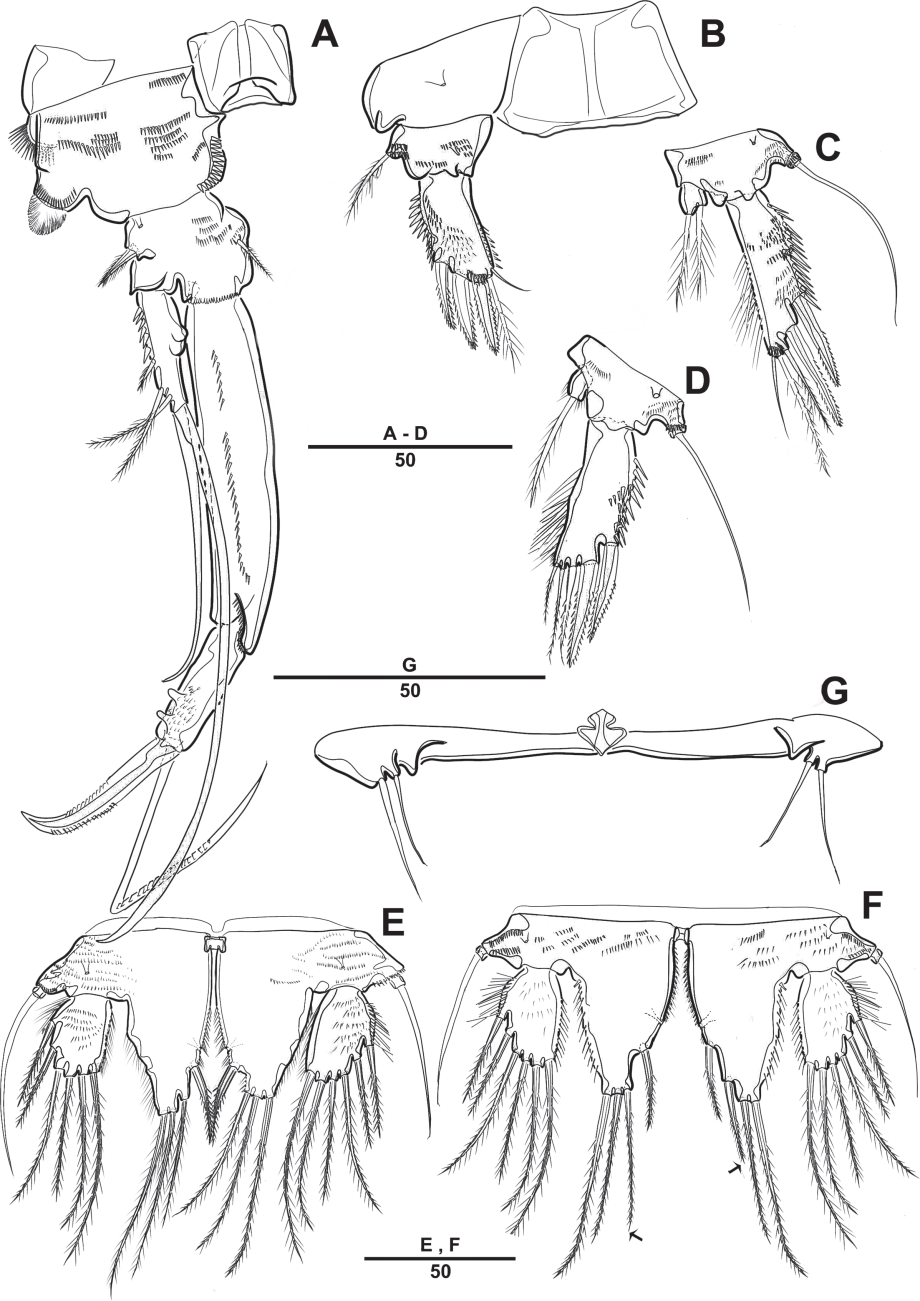
*Intercristacoxa koreana* sp. nov., ♀, MABIK CR00258582. **A.** P1; **B.** P2; **C.** P3; **D.** P4; **E.** P5; **F.** Abnormality in P5 (MABIK CR00258581); **G.** P6. Scale bars in μm.

**Figure 15.**
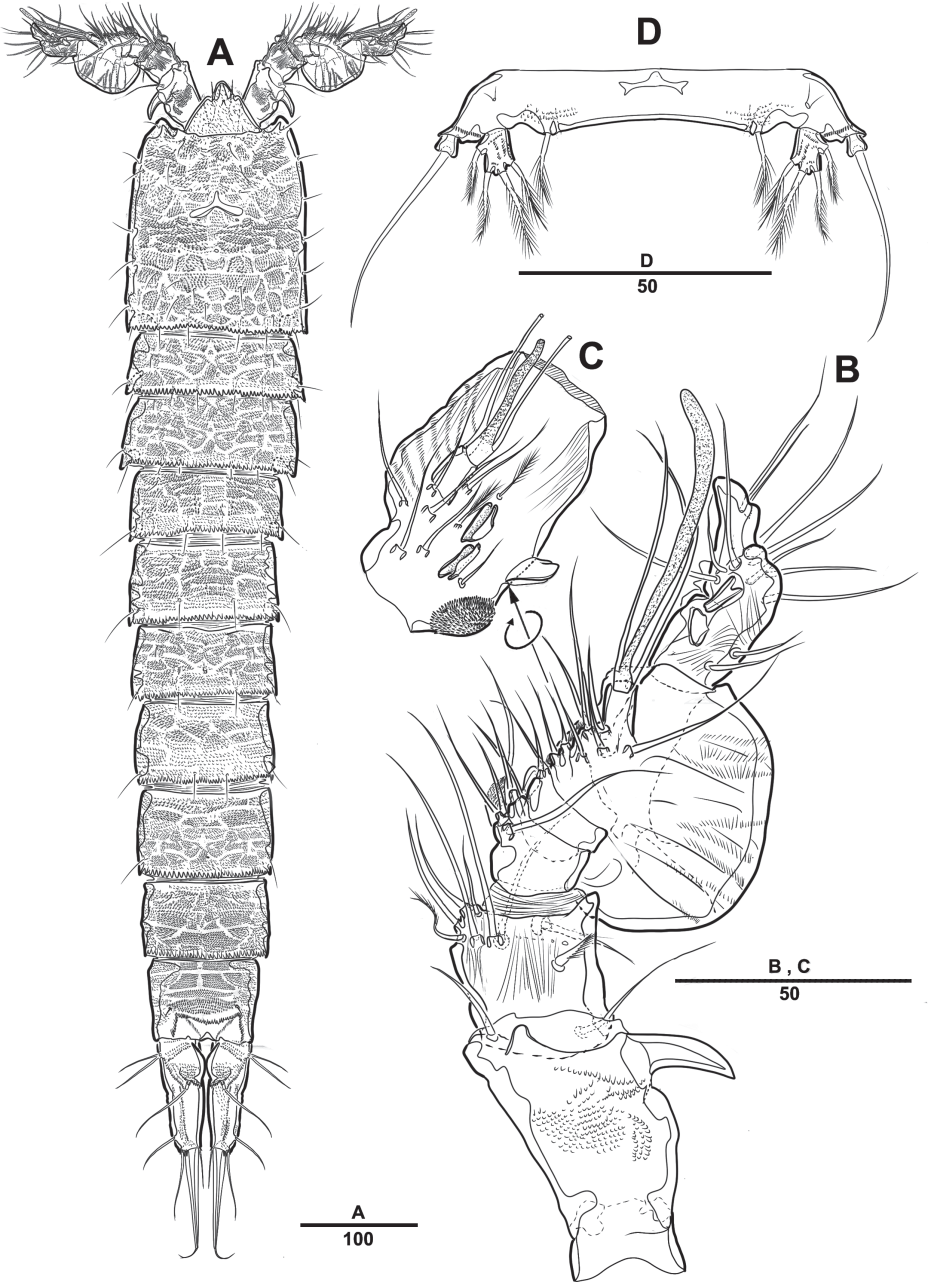
*Intercristacoxa koreana* sp. nov., ♂, MABIK CR00258579. **A.** Habitus, dorsal; **B.**A1; **C.**A1seg–5; **D.** P5. Scale bars in μm.

**Figure 16.**
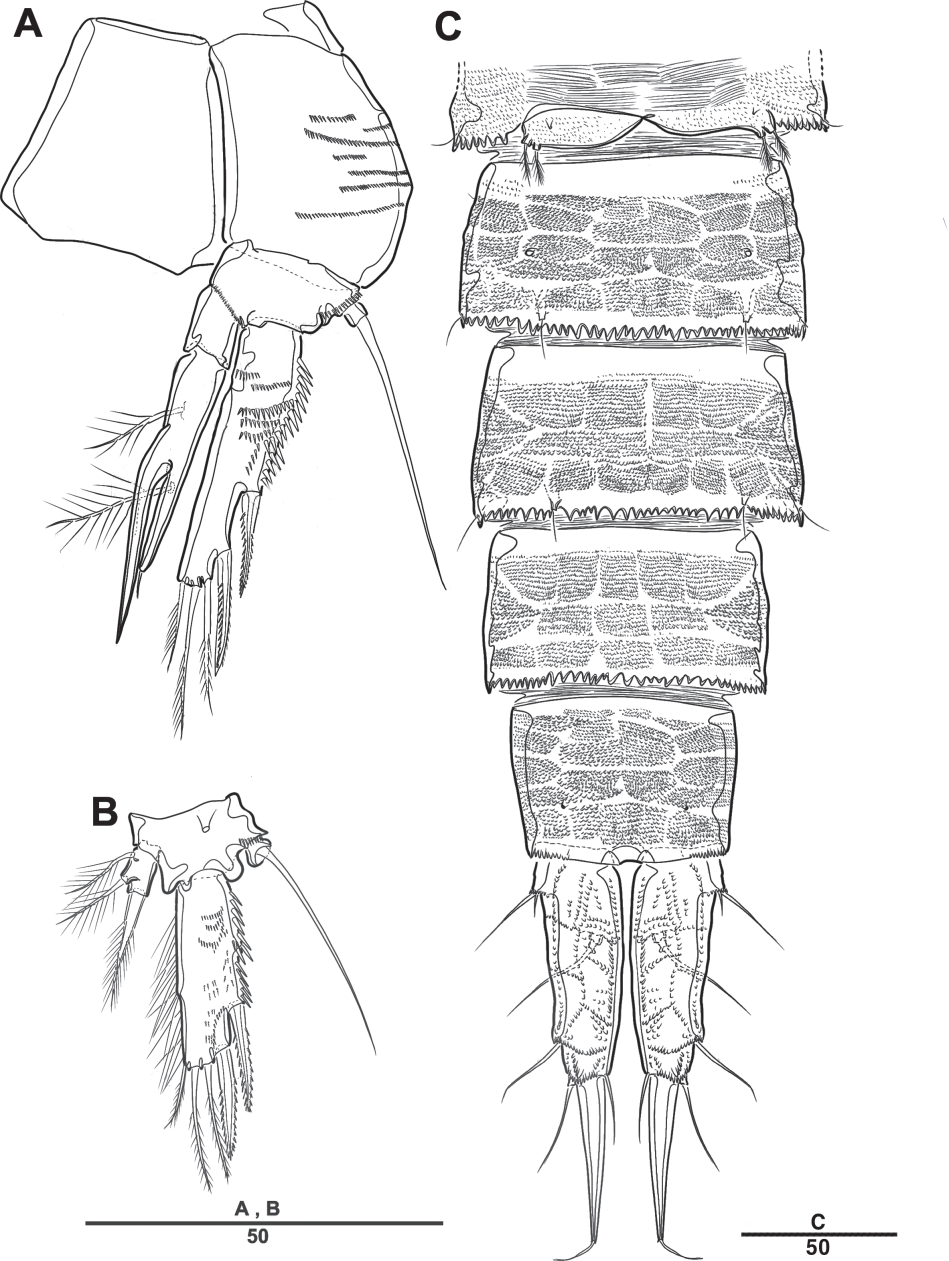
*Intercristacoxa koreana* sp. nov., ♂, MABIK CR00258579. **A.** P3; **B.** P4; **C.** Urosome; and P6. Scale bars in μm.

**Figure 17.**
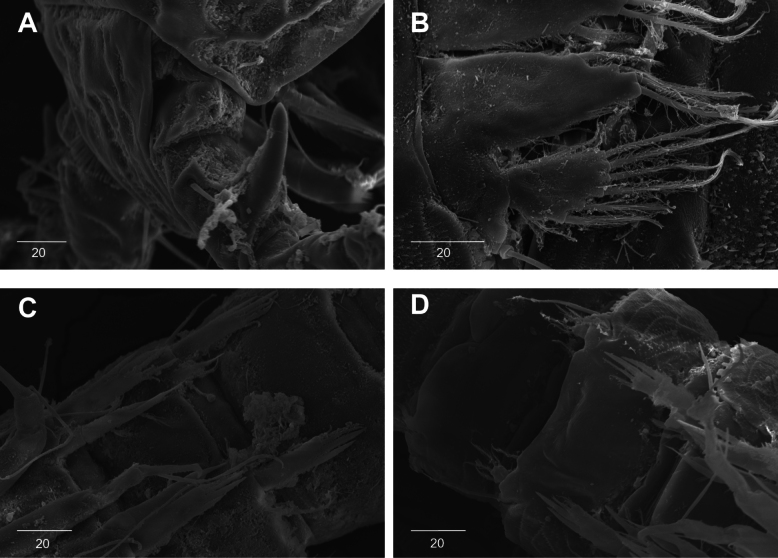
*Intercristacoxa koreana* sp. nov., **A.** ♀, SEM; A1seg–1; **B.** ♀, SEM, P5; **C.** ♂, SEM, P3 and P4; **D.** ♂, SEM, P5. Scale bars in μm.

Figs 11, 12, 13, 14, 15, 16, 17

##### Type locality.

Subtidal, site B (37°14'23.23"N, 131°52'20.06"E, Fig. 1), depth 7–10 m, sand between cracks in stone, May 16, 2017.

##### Type material.

***Holotype***: • 1♀ (MABIK CR00258582), dissected on ten slides. ***Paratypes***: • 1♂ (MABIK CR00258579), dissected on nine slides; • 1♀ (MABIK CR00258581), dissected on nine slides; • 1♂ (MABIK CR00258580), dissected on five slides. Three specimens (1♀, 2♂♂) dried, mounted on stub, and coated with gold for SEM.

##### GenBank accession number.

Mitochondrial cytochrome c oxidase subunit I genes (PX210748–PX210750, PX485050–PX485054), 18S ribonucleic acid genes (PX218726, PX218727, PX485043–PX485045).

##### Description.

Female. Total body (Fig. 11A, B), nearly cylindrical, with minute sensilla dorsally, length from anterior margin of rostrum to posterior margin of caudal rami 920 μm, maximum width 160 μm measured at end of cephalothorax; rostrum (Fig. 11C) well developed, defined at base, triangular, with two sensilla; cephalothorax wider than free somites, pleural areas of cephalic shield narrow and posterolateral angles rounded; second and third urosomites (genital double somite) separated dorsally and laterally, fused ventrally but discontinuous internal chitinous rib indicating original segmentation (Figs 11B, 12C), genital apparatus located at anterior of genital double somite; anal operculum deeply curved (Fig. 12D).

Caudal rami (Fig. 12C, D), parallel, rectangular, ~ 1.3 × longer than maximum width; each ramus with seven setae: seta I minute; seta II bare, laterally; seta III bare, located near distal corner; seta IV bare; seta V bi-articulated, consists of spine-like seta at top and small naked seta attached at end; seta VI bare at inner distal corner; seta VII tri-articulated at base, bare.

A1 (Figs 12A, 17A): four-segmented; seg–1 with one thorn-like process at distal outer edge; seg–3 longest, with sub-cylindrical pedestal with ae fused at base to one naked seta; armature formula: 1–[2], 2–[9], 3–[9 + (1 + ae)], 4–[14]; apical ae not observed, but two apical long setae fused at base.

A2 (Fig. 12B): three-segmented; coxa bare; allobasis without abexopodal seta; exp one-segmented with two small setae; free enp with four spines, three geniculate setae, and one bare seta.

Labrum (Fig. 13A) with a dense pattern of blunt processes in middle and numerous spinules of overlapping scales.

Md (Fig. 13B): gnathobase developed, with one uni-pinnate seta laterally and several teeth; basis of palp with one bare seta along inner margin; enp one-segmented, with three bare apical setae; exp represented by one bare seta.

Mxl (Fig. 13C): praecoxa trapezoidal; arthrite well developed with two juxtaposed bare setae on anterior surface, two pinnate setae laterally, and six elements around distal margin; coxa with one bare and one pinnate seta; basis with four setae; enp incorporated in basis, represented by small protrusion with three bare setae; exp one-segmented, with two bare setae.

Mxa (Fig. 13D): syncoxa with three endites; proximal endite vestigial, represented by one seta; second endite with one strong spine and two bare setae; distal endite with three setae; allobasis produced into strong curved claw, accessory armature consisting of two setae; and enp represented by three bare setae fused at base.

Mxp (Fig. 13E): three-segmented; syncoxa with one pinnate seta; basis slender, elongate, and bare; enp represented by an acutely recurved claw with two accessory setae.

P1 (Fig. 14A): coxa with two outer serrate crests; basis with one outer and one inner pinnate setae, ornamented with small frills on base of enp; exp one-segmented, with three outer and two apical setae, ornamented with two protuberances; enp two-segmented, prehensile; enp–1 elongate, unarmed; enp–2 rectangular, with one curved claw and one geniculate seta.

P2 (Fig. 14B): coxa bare; basis with one outer pinnate seta; exp one-segmented, with three spines and two setae; enp absent.

P3 (Fig. 14C): coxa bare; basis with one outer bare seta; exp one-segmented, with two outer spines, one bare seta, and two pinnate setae; and enp one-segmented, with two pinnate setae.

P4 (Fig. 14D): coxa bare; basis with one outer bare seta; exp one-segmented, with two outer spines and three pinnate setae; enp one-segmented, with one pinnate seta.

Armature formulae as follows:

**Table:** 

	Exp	Enp
P2	113	–
P3	122	011
P4	122	010

P5 (Figs 14E, 17B): basal part with one outer bare seta; endopodal lobes not fused medially, connected by intercoxal sclerite, with four pinnate setae, an abnormality observed in second innermost seta of paratype (Fig. 14F, black arrow); exp defined at base, with six pinnate setae.

P6 (Fig. 14G): P6 vestigial, represented by a small bump with two bare setae.

Male. Total body (Fig. 15A) length from anterior margin of rostrum to posterior margin of caudal rami 890 μm, maximum width 155 μm measured at midway of cephalothorax.

General body shape, ornamentation, and sensilla pattern same as in females, except for genital double somites and sexual dimorphism in A1, P3, P4, P5, and P6.

A1 (Fig. 15B, C): six-segmented, robust, chirocer; seg–1 with one thorn-like process at distal outer edge; seg–5 swollen, with pad-like process near anterior margin, three processes in middle, and ae fused at base to one bare seta, armature formula: 1–[1], 2–[10], 3–[6], 4–[2], 5–[9 + (1 + ae)], 6–[12], apical ae not observed.

Setal formula and general shape of P1 and P2 similar to those of female.

P3 (Figs 16A, 17C): coxa bare; basis with one outer bare seta; exp one-segmented, with two spines and three setae; enp two-segmented; enp–1 small, bare; enp–2 elongated, armed with spinous apophysis, with two lateral pinnate setae, and one apical minute seta.

P4 (Figs 16B, 17C): coxa bare; basis with one outer bare seta; exp one-segmented, with two spines and three setae, ornamented with setules along inner margin; enp one-segmented, with one apical (stouter than female) and two lateral pinnate setae.

P5 (Figs 15D, 17D): benp represented by a small protrusion with one pinnate seta; exp defined at base, with four pinnate setae.

P6 (Fig. 16C): asymmetrical, with functional right member articulating at base and closing off genital aperture, and left member fused at base to genital somite; each P6 with two pinnate setae.

##### Etymology.

The species name refers to the country of the type locality.

#### ﻿ Intercristacoxa trisetosa sp. nov.

Taxon classificationAnimaliaHarpacticoidaOrthopsyllidae

28C98F05-645A-5617-BC4B-8D796F3708A5

https://zoobank.org/C443816B-365F-47CE-AEBA-9767890DABBE

Figs 18, 19, 20, 21, 22, 23, 24, 25, 26

##### Type locality.

Subtidal, site C (35°55'30.6"N, 129°32'52.3"E, Fig. 1), depth 10 m, sand between cracks in stone, on April 11.

##### Type material.

***Holotype***: • 1♀ (MABIK CR00258665), dissected on thirteen slides. ***Paratypes***: • 1♀ (MABIK CR00258666), dissected on ten slides; • 1♂ (MABIK CR00258667), dissected on nine slides.

##### GenBank accession number.

Mitochondrial cytochrome c oxidase subunit I gene (PX210753); 18S ribonucleic acid genes (PX218730 and PX218731).

##### Description.

**Figure 18.**
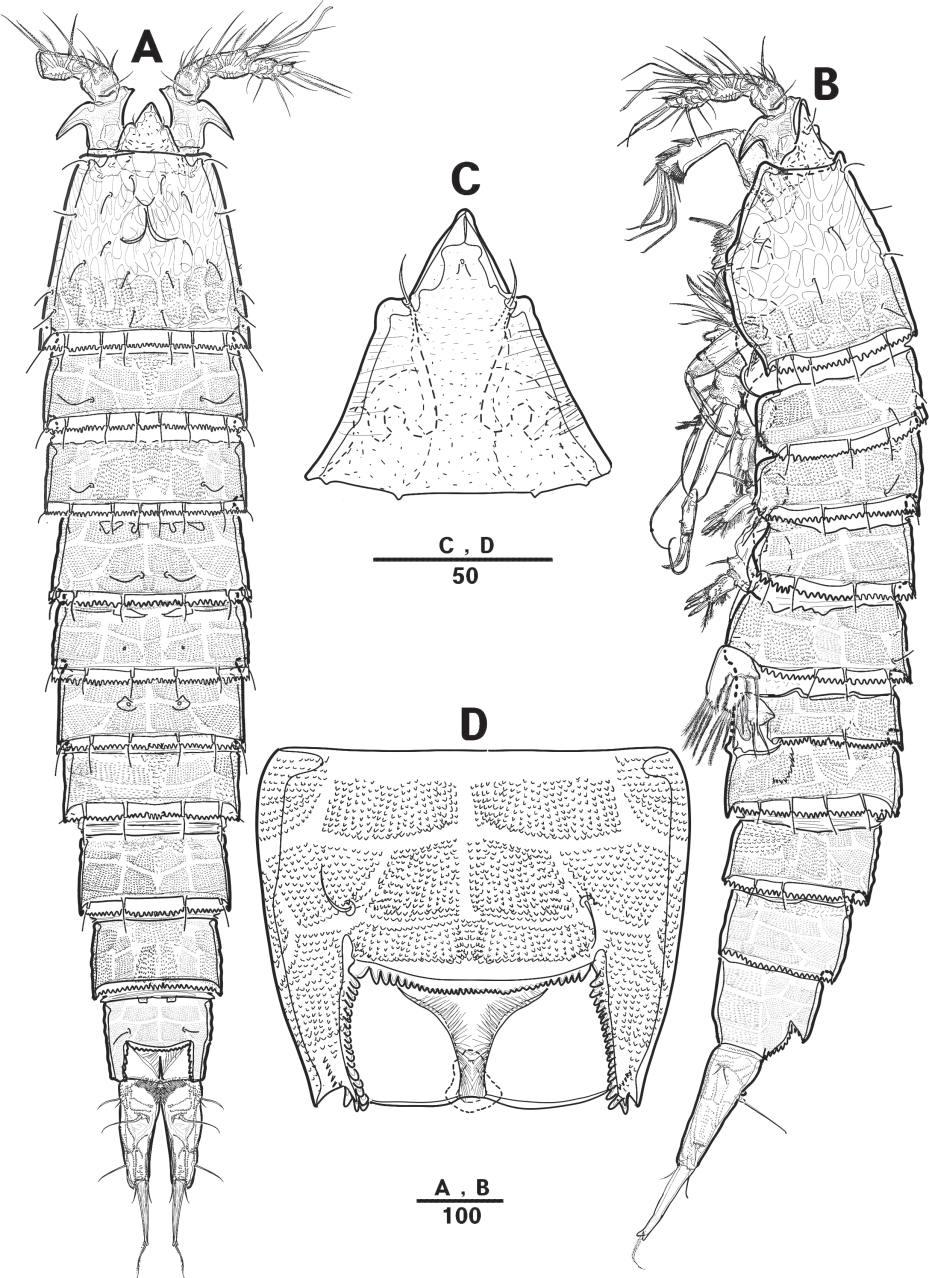
*Intercristacoxa trisetosa* sp. nov., ♀, MABIK CR00258665. **A.** Habitus, dorsal; **B.** Habitus, lateral; **C.** Rostrum; **D.** Anal somite. Scale bars in μm.

Female. Total body (Fig. 18A, B), nearly cylindrical, with minute sensilla dorsally, length from anterior margin of rostrum to posterior margin of caudal rami 1,260 μm, maximum width 240 μm measured at end of cephalothorax; rostrum (Fig. 18C) well developed, defined at base, tri-angular, with two sensilla; cephalothorax wider than free somites, pleural areas of cephalic shield narrow and posterolateral angles rounded; second and third urosomites (genital double somite) separated dorsally and laterally, fused ventrally but discontinuous internal chitinous rib indicating original segmentation, genital apparatus located at anterior of genital double somite (Fig. 22A); anal operculum deeply curved (Fig. 18D).

Caudal rami (Figs 22A, 23A–C): parallel; each ramus with seven setae; seta V bi-articulated, consists of spine-like seta at top and small pinnate seta attached at end (Fig. 23B); seta VI bare at inner distal corner; seta VII tri-articulated at base.

**Figure 19.**
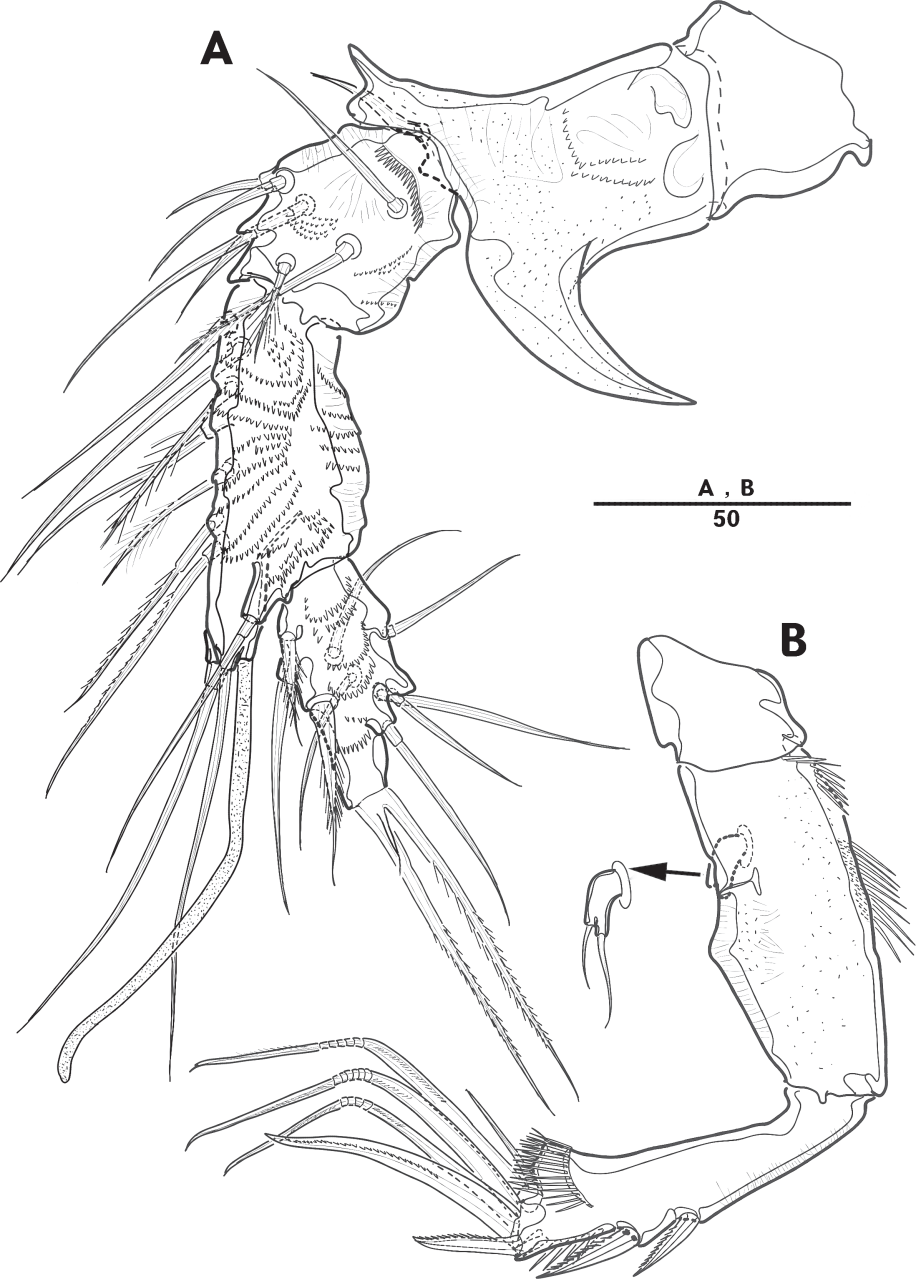
*Intercristacoxa trisetosa* sp. nov., ♀, MABIK CR00258665. **A.**A1; **B.**A2. Scale bars in μm.

A1 (Fig. 19A): four-segmented; seg–1 with one thorn-like process at distal outer edge; seg–3 longest, with one ae; armature formula: 1–[1], 2–[7], 3–[10 + ae], 4–[12]; apical ae not observed, but three apical setae fused at base.

A2 (Fig. 19B): three-segmented; coxa bare; allobasis without abexopodal seta; exp one-segmented with two setae; free enp with four spines, three geniculate setae, and one bare seta.

**Figure 20.**
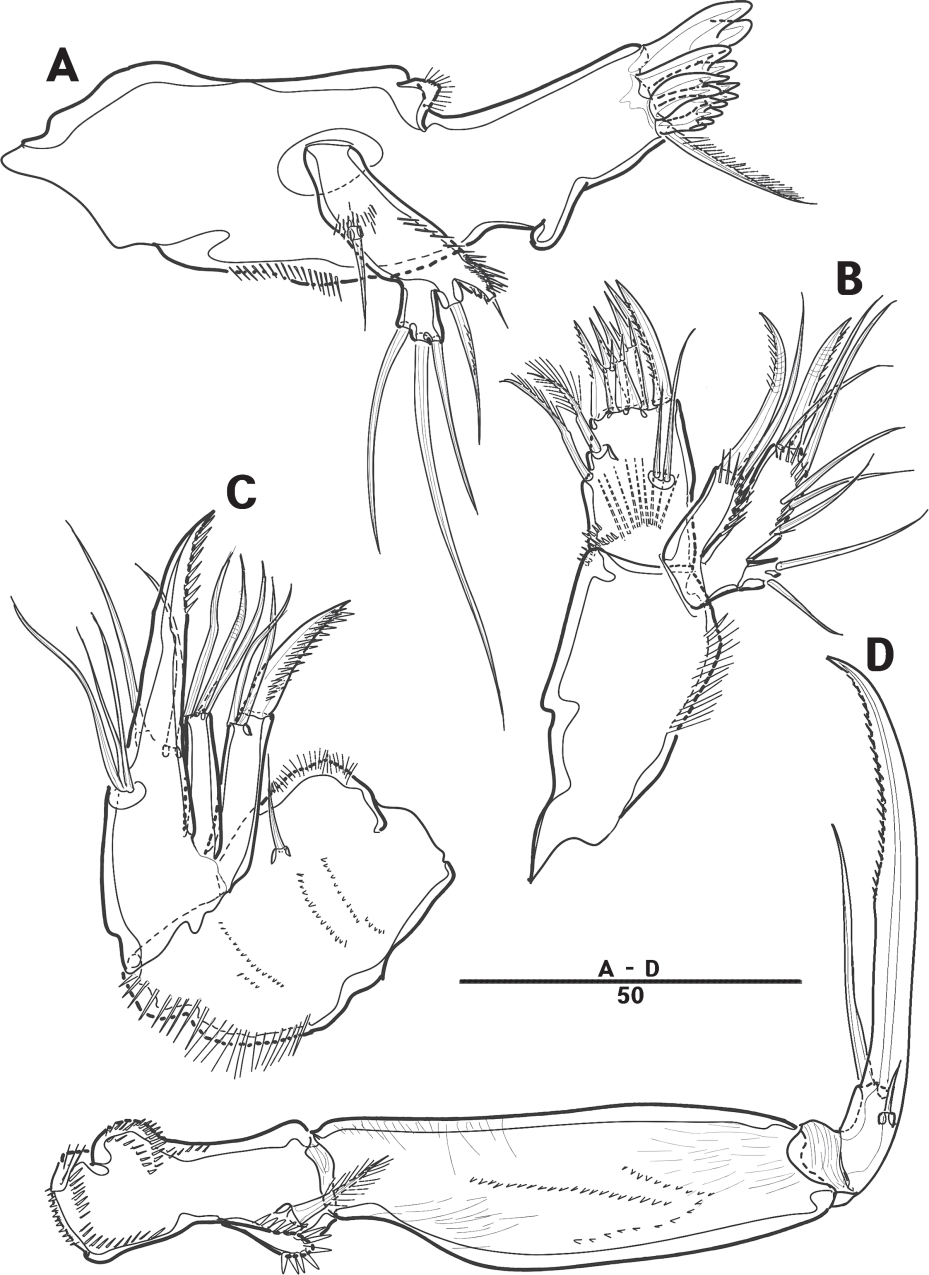
*Intercristacoxa trisetosa* sp. nov., ♀, MABIK CR00258665. **A.**Md; B. Mxl; **C.**Mxa; **D.**Mxp. Scale bars in μm.

Md (Fig. 20A): gnathobase with one uni-pinnate seta laterally and several teeth; basis of palp with two setae; enp one-segmented with three bare setae; exp incorporated into basis, represented by small protrusion with one bare seta.

Mxl (Fig. 20B): praecoxa trapezoidal; arthrite well developed with two juxtaposed bare setae on anterior surface, two pinnate setae laterally, and six elements around distal margin; coxa with one spine and one seta; basis with one spine and three setae; enp incorporated in basis, represented by small protrusion with three bare setae; exp one-segmented, with two setae.

Mxa (Fig. 20C): syncoxa with three endites; proximal endite vestigial, represented by one seta; second endite with one strong spine and two bare setae; distal endite with one geniculate and two bare setae; allobasis produced into strong curved claw, accessory armature consisting of two setae; and enp represented by three bare setae fused at base.

Mxp (Fig. 20D): three-segmented; syncoxa with one pinnate seta; basis slender, elongate, and bare; enp represented by an acutely recurved claw with two accessory setae.

**Figure 21.**
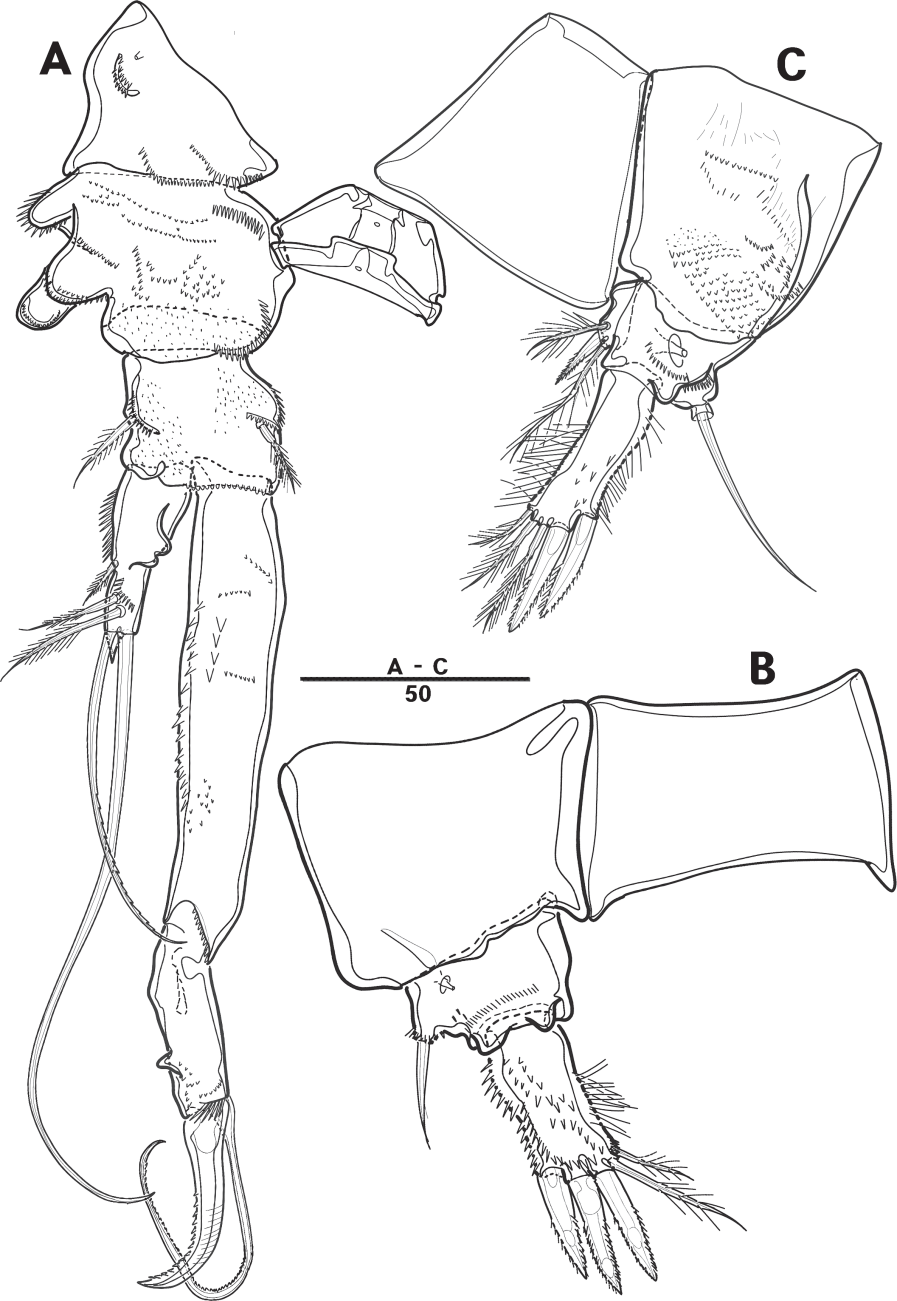
*Intercristacoxa trisetosa* sp. nov., ♀, MABIK CR00258665. **A.** P1; **B.** P2; **C.** P3. Scale bars in μm.

P1 (Fig. 21A): coxa with two outer serrate crests; basis with one outer and one inner pinnate setae, ornamented with small frills on base of enp; exp one-segmented, with three outer and two distal setae, ornamented with two small protuberances; enp two-segmented, prehensile; enp–1 elongate, unarmed; enp–2 rectangular, with one curved claw and one seta.

**Figure 22.**
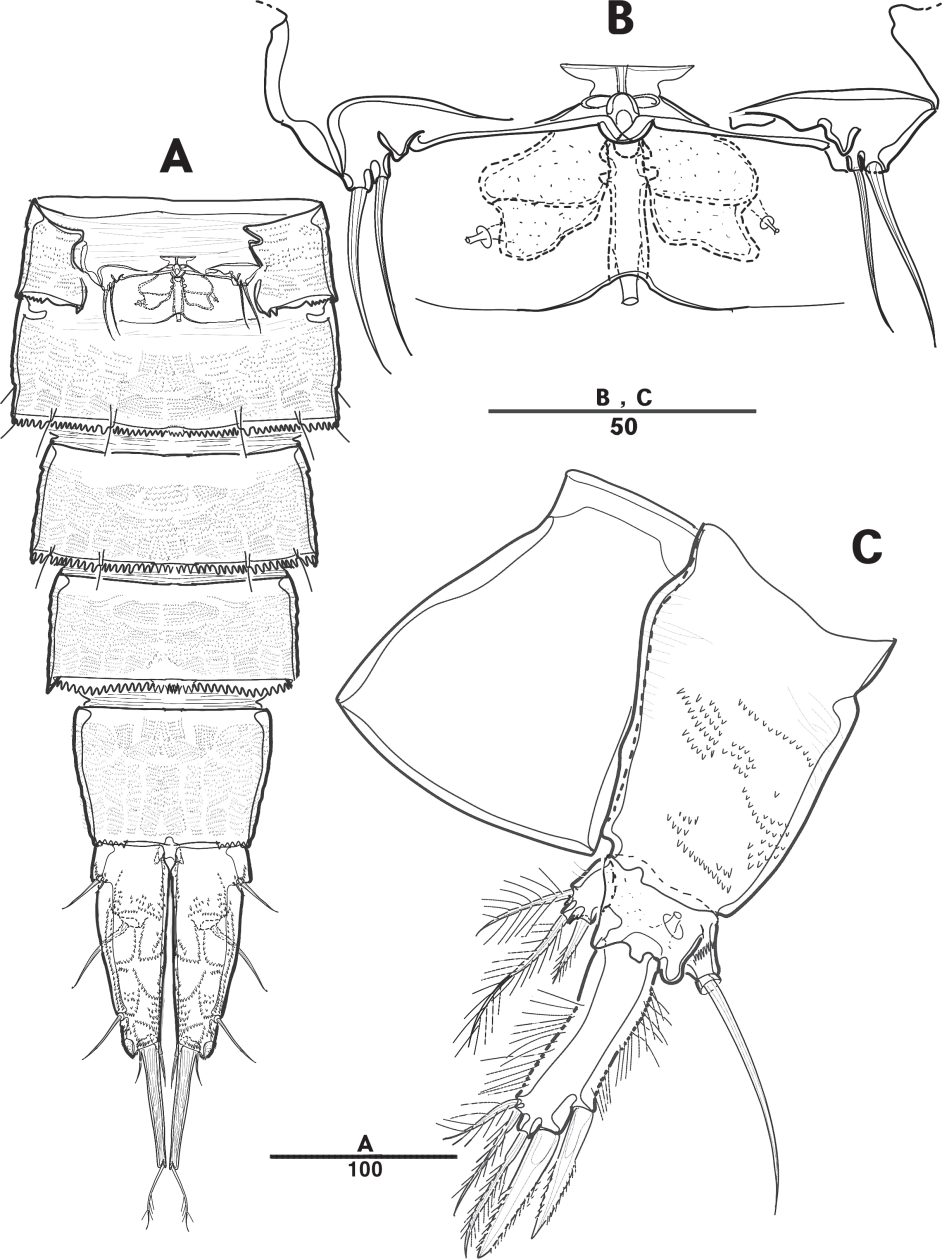
*Intercristacoxa trisetosa* sp. nov., ♀, MABIK CR00258665. **A.** Urosome, ventral; **B.** Genital field and P6; **C.** P4. Scale bars in μm.

**Figure 23.**
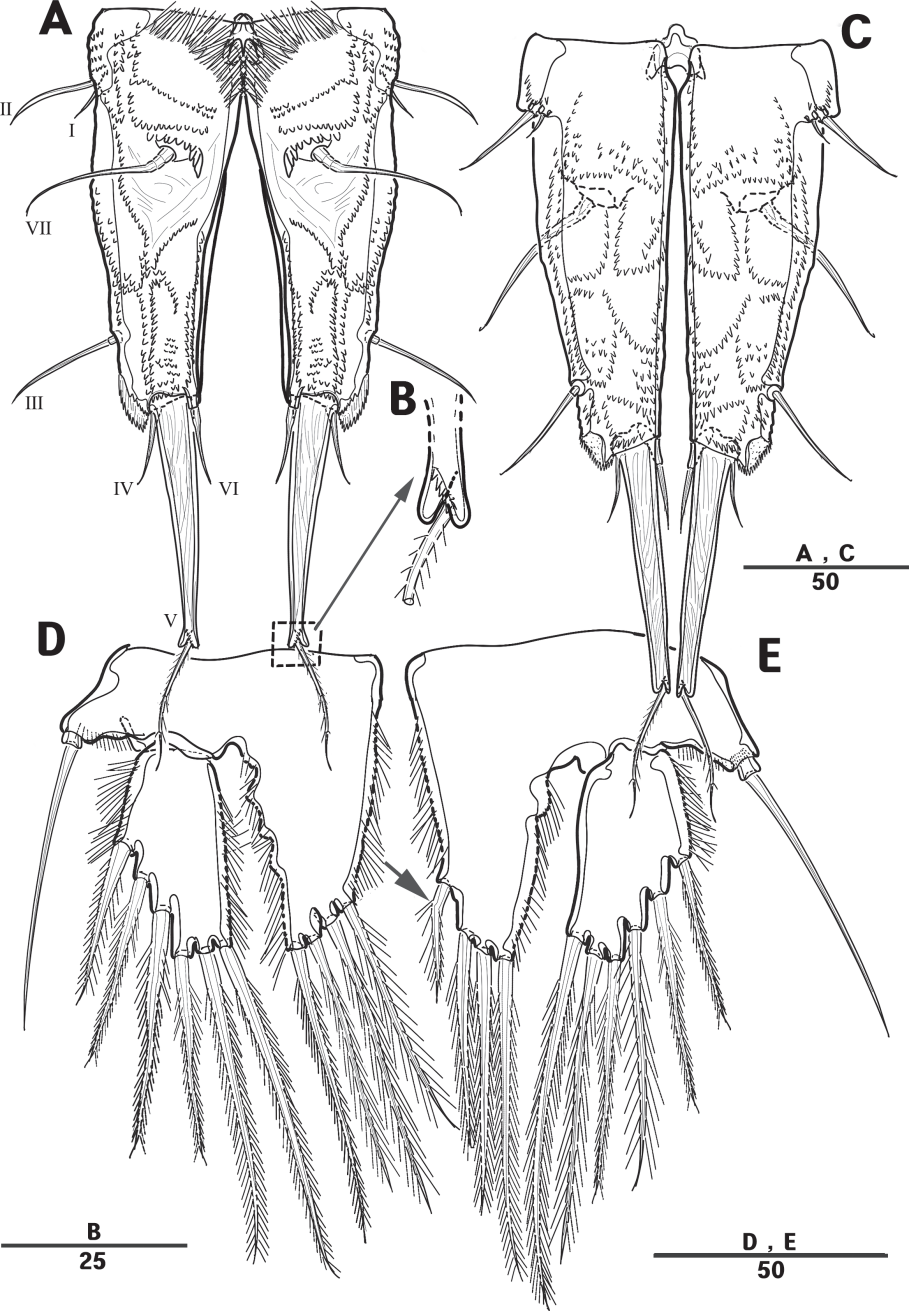
*Intercristacoxa trisetosa* sp. nov., ♀, MABIK CR00258665. **A.** Caudal rami, dorsal; **B.** Caudal seta V, bi-articulated; **C.** Caudal rami, ventral; **D.** P5; **E.** Abnormality in P5 (MABIK CR00258666). Scale bars in μm.

P2 (Fig. 21B): coxa bare; basis with one outer pinnate seta; exp one-segmented, with three spines and two pinnate setae; enp absent.

P3 (Fig. 21C): coxa bare, basis with one outer bare seta; exp one-segmented with two outer spines and three pinnate setae; enp one-segmented with three setae.

P4 (Fig. 22C): coxa bare; basis with one outer bare seta; exp one-segmented, with two spines and three pinnate setae, ornamented with a row of setules along inner and outer margins; enp one-segmented, with three setae.

Armature formulae as follows:

**Table:** 

	Exp	Enp
P2	113	–
P3	122	111
P4	122	111

P5 (Fig. 23D): basal part with one outer bare seta; endopodal lobe with four pinnate setae, an abnormality observed in innermost seta of paratype (Fig. 23E, black arrow); exp defined at base with six pinnate setae.

P6 (Fig. 22B): P6 vestigial, represented by a small bump with two bare setae.

**Figure 24.**
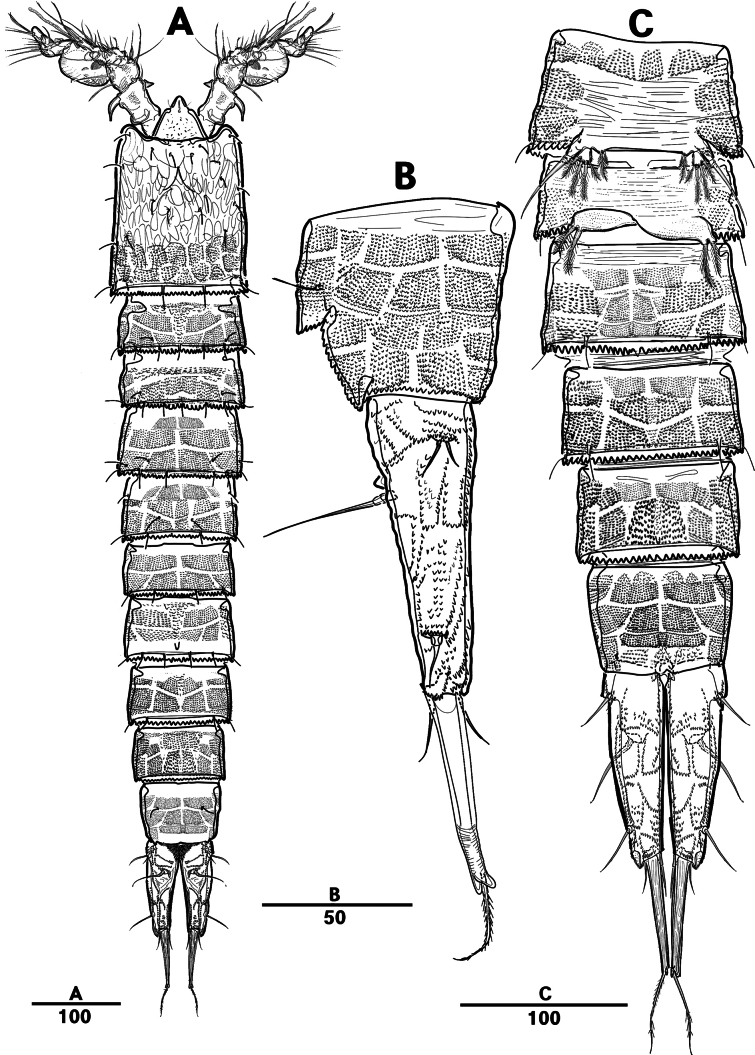
*Intercristacoxa trisetosa* sp. nov., ♂, MABIK CR00258667. **A.** Habitus, dorsal; **B.** Anal somite and caudal rami, lateral; **C.** Urosome and P6. Scale bars in μm.

Male. Total body (Fig. 24A): length from anterior margin of rostrum to posterior margin of caudal rami 945 μm; maximum width 150 μm measured at midway of cephalothorax.

General body shape, ornamentation, and sensilla pattern same as in females, except for genital double somites and sexual dimorphism in A1, P3, P4, P5, and P6.

**Figure 25.**
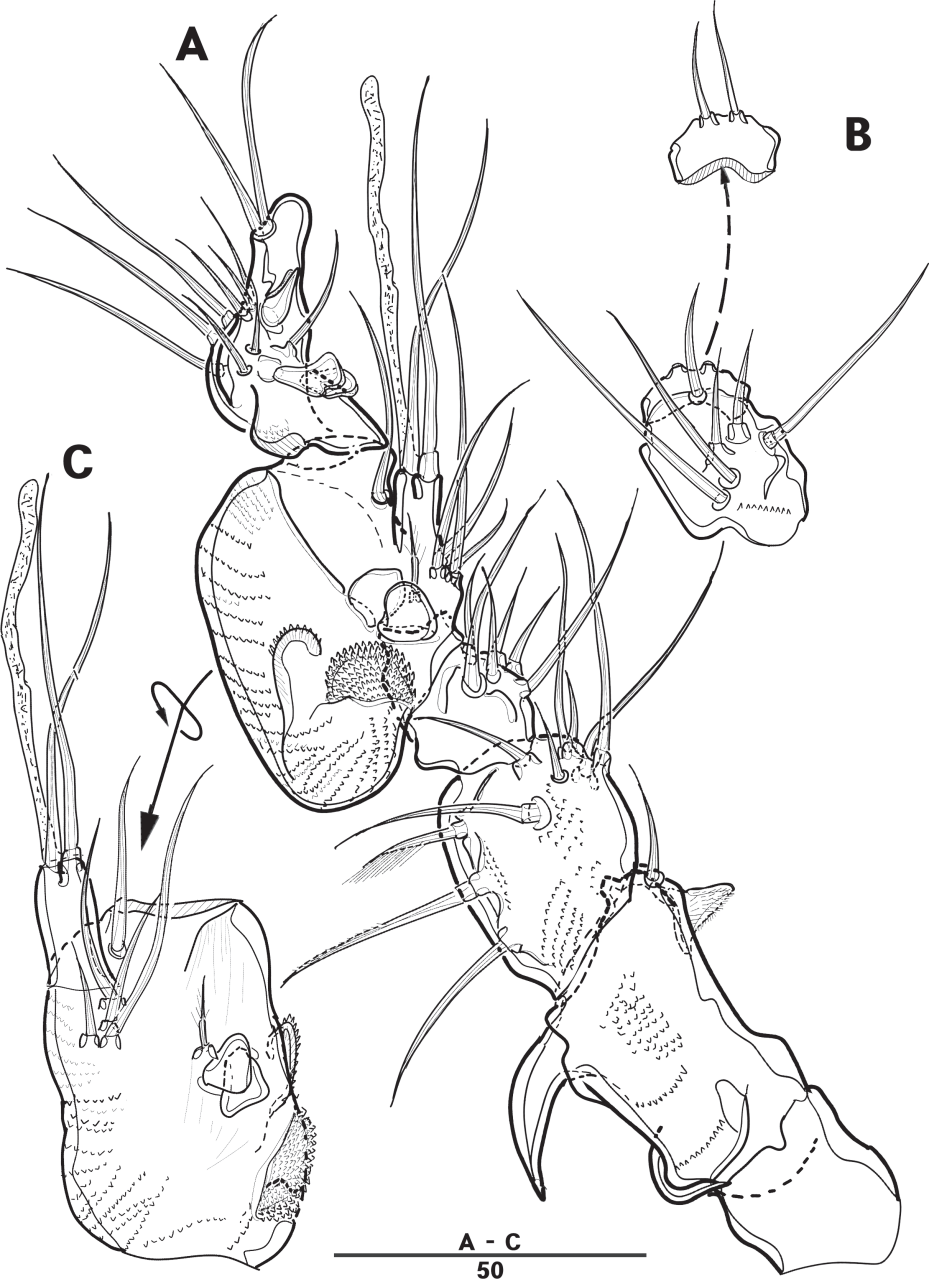
*Intercristacoxa trisetosa* sp. nov., ♂, MABIK CR00258667. **A.**A1; **B.** Seg–4 and –5 of A1; **C.** Seg–5 of A1.

A1 (Fig. 25A–C): six-segmented, robust, chirocer; seg–1 with one thorn-like process at distal outer edge; seg–5 swollen, with pad-like process near anterior margin, three processes in middle, and ae fused at base to one bare seta, armature formula: 1–[1], 2–[10], 3–[6], 4–[2], 5–[7 + (1 + ae)], 6–[12], apical ae not observed.

**Figure 26.**
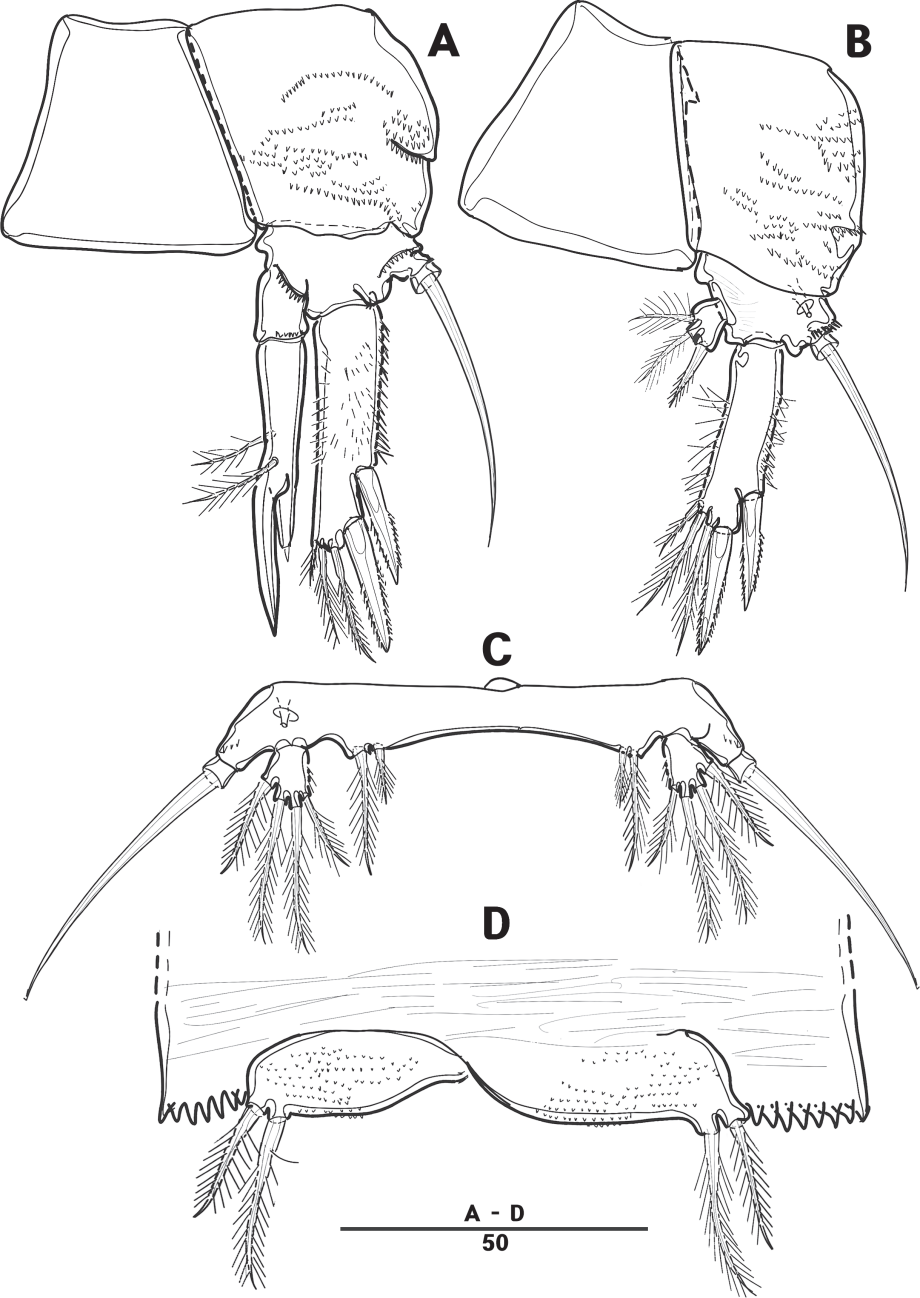
*Intercristacoxa trisetosa* sp. nov., ♂, MABIK CR00258667. **A.** P3; **B.** P4; **C.** P5; **D.** P6. Scale bars in μm.

P3 (Fig. 26A): coxa bare; basis with one outer bare seta; exp one-segmented, with two spines and three setae; enp two-segmented; enp–1 small, bare; enp–2 elongated, armed with a spinous apophysis, with two lateral pinnate and one apical minute setae.

P4 (Fig. 26B): coxa bare, basis with one outer bare seta; exp one-segmented, with two spines and three setae; enp one-segmented, with three setae (middle setae shorter than in females).

P5 (Fig. 26C): benp represented by small protrusion with two pinnate setae; exp defined at base, with four pinnate setae.

P6 (Fig. 26D): asymmetrical, with functional right member articulating at base and closing off genital aperture, and left member fused at base to genital somite; each P6 with two pinnate setae.

##### Etymology.

The species name refers to the character of the P3 and P4 enp armed with three setae.

### ﻿Genus *Orthopsyllus* Brady & Robertson, 1873

#### ﻿ Orthopsyllus ulsanus sp. nov.

Taxon classificationAnimaliaHarpacticoidaOrthopsyllidae

30486336-6BDA-59CC-904F-B526268F74F4

https://zoobank.org/40F0BA2D-6750-45E1-9D85-5DD1013D9DA6

Figs 27, 28, 29, 30, 31, 32, 33, 34, 35, 36

##### Type locality.

Subtidal, site D (35°29'27.67"N, 129°26'34.48"E, Fig. 1) using a grab sampler. Paratype was collected from site E (34°24'58.9"N, 127°54'20.4"E, Fig. 1).

##### Type material.

***Holotype***: • 1♀ (MABIK CR00259122) dissected on ten slides. ***Paratype***: • 1 ♂ (MABIK CR00259121) dissected on eight slides.

##### GenBank accession number.

Mitochondrial cytochrome c oxidase subunit I genes (PX210754, PX210755, PX485059–PX485064); 18S ribonucleic acid genes (PX218732, PX218733, PX485046–PX485049).

##### Description.

**Figure 27.**
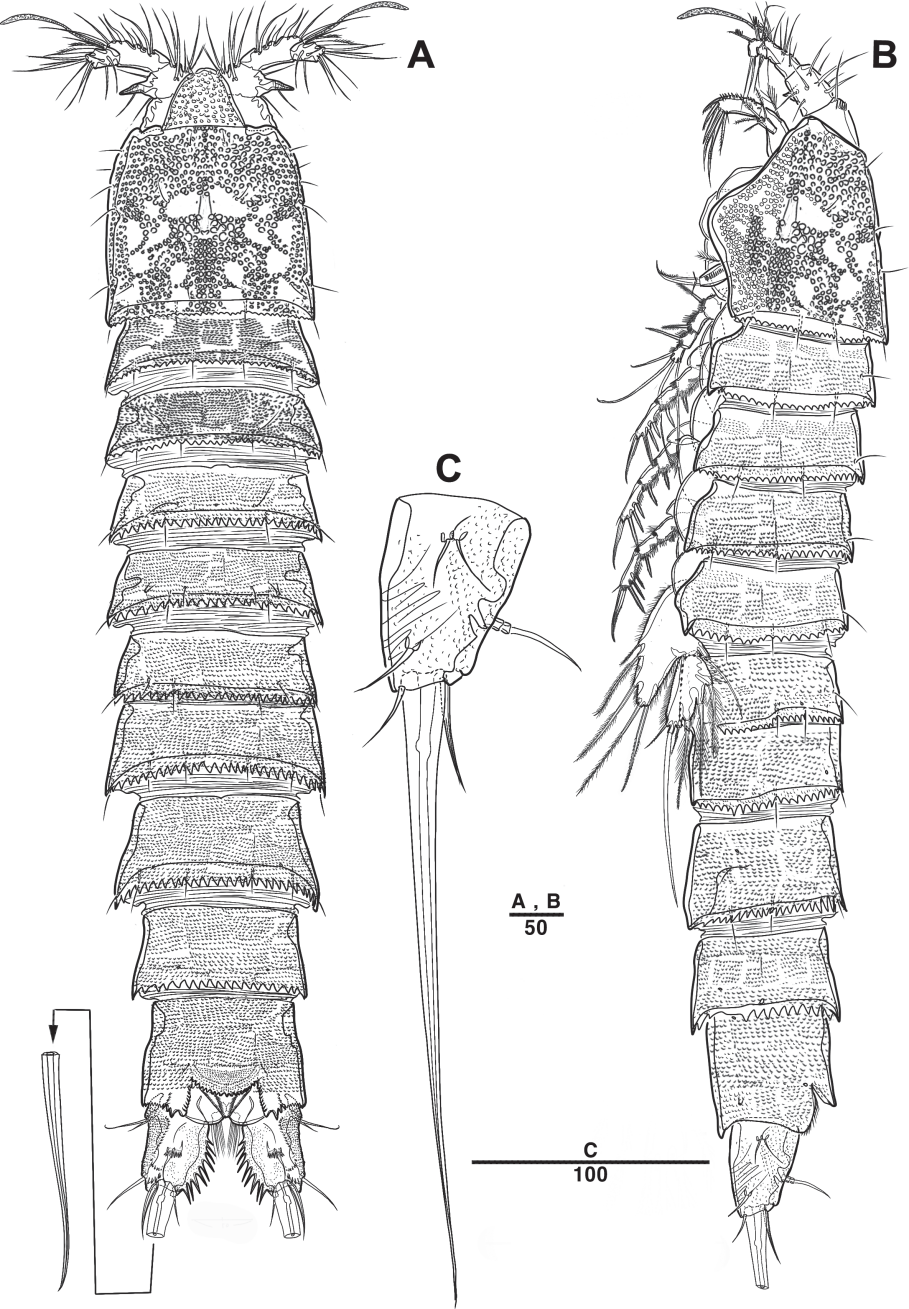
*Orthopsyllus ulsanus* sp. nov., ♀, MABIK CR00259122. **A.** Habitus, dorsal; **B.** Habitus, lateral; **C.** Caudal rami, lateral. Scale bars in μm.

Female. Total body (Fig. 27A, B), length from anterior margin of rostrum to posterior margin of caudal rami 1,040 μm, maximum width 170 μm measured at end of cephalothorax.

Body (Fig. 27A, B): cylindrical and not dorsoventrally depressed, with minute sensilla dorsally; rostrum well developed, defined at base, triangular, smooth at tip, with two sensilla; cephalothorax wider than free somites, several pits scattered on surface (Fig. 36A), pleural areas of cephalic shield narrow and posterolateral angles rounded; second and third urosomites (genital double somite) separated dorsally and laterally fused ventrally but discontinuous internal chitinous rib indicating original segmentation (Fig. 27B and Fig. 29C), genital apparatus located mid-ventrally at anterior half of genital double somite, genital pores covered by P6; complex of seminal receptacles paired, transparent cuticle (Fig. 29B, C); anal operculum deeply curved, serrated (Fig. 29D).

Caudal rami (Figs 27C, 29C, D, 36F): parallel, rectangular, approximately 1.3 × longer than maximum width; inner lamella ornamented with nine teeth; each ramus with seven setae: seta V normal, longest; seta VI bare at inner distal corner, longer than seta IV; seta VII bare, tri-articulated, all setae bare.

**Figure 28.**
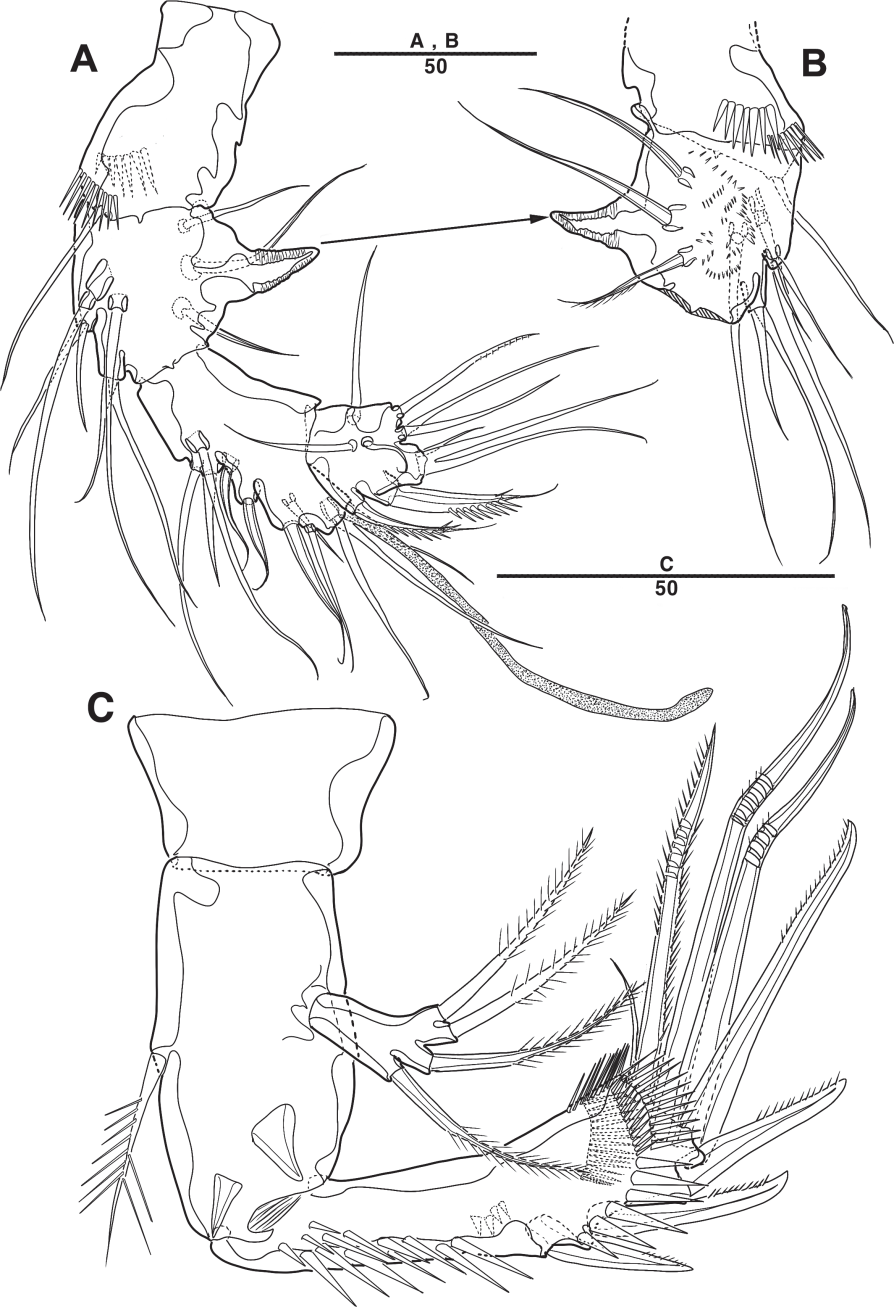
*Orthopsyllus ulsanus* v, ♀, MABIK CR00259122. **A.**A1; **B.** Seg–1 and –2 of A1; **C.**A2. Scale bars in μm.

A1 (Fig. 28A, B): four-segmented; seg–2 with a wrinkled process; seg–3 longest, with sub-cylindrical pedestal with ae fused at base to one naked seta; armature formula: 1–[1], 2–[9], 3–[10 + (1 + ae)], 4–[11]; apical ae not observed, but two apical long setae fused at base.

**Figure 29.**
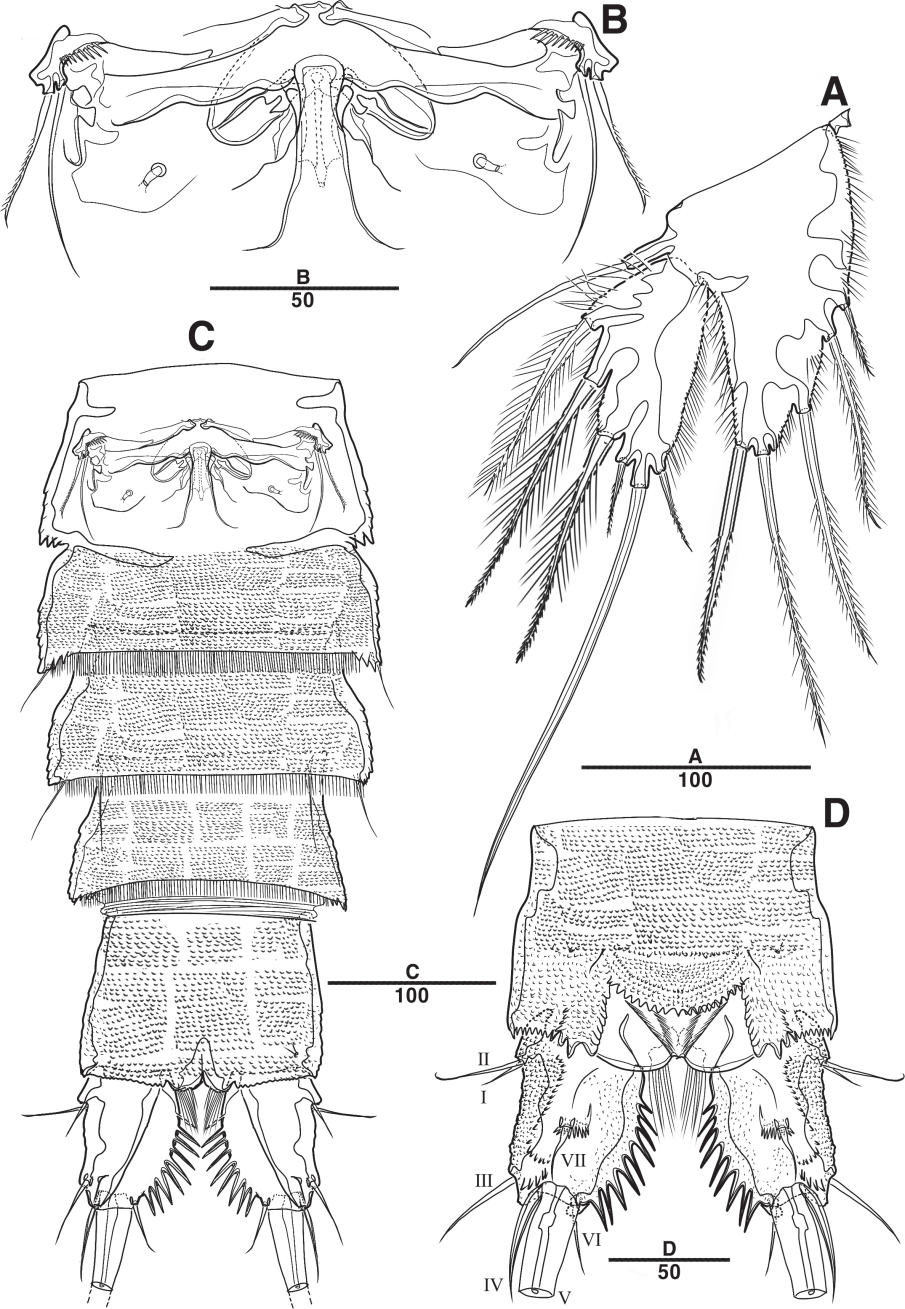
*Orthopsyllus ulsanus* sp. nov., ♀, MABIK CR00259122. **A.** P5; **B.** P6 and genital field; **C.** Urosome, dorsal; **D.** Anal somite and caudal rami, dorsal. Scale bars in μm.

A2 (Fig. 28C): three-segmented; coxa bare; allobasis with one abexopodal pinnate seta; exp one-segmented, with four pinnate setae; free enp with four spines, three geniculate setae, and one bare seta.

**Figure 30.**
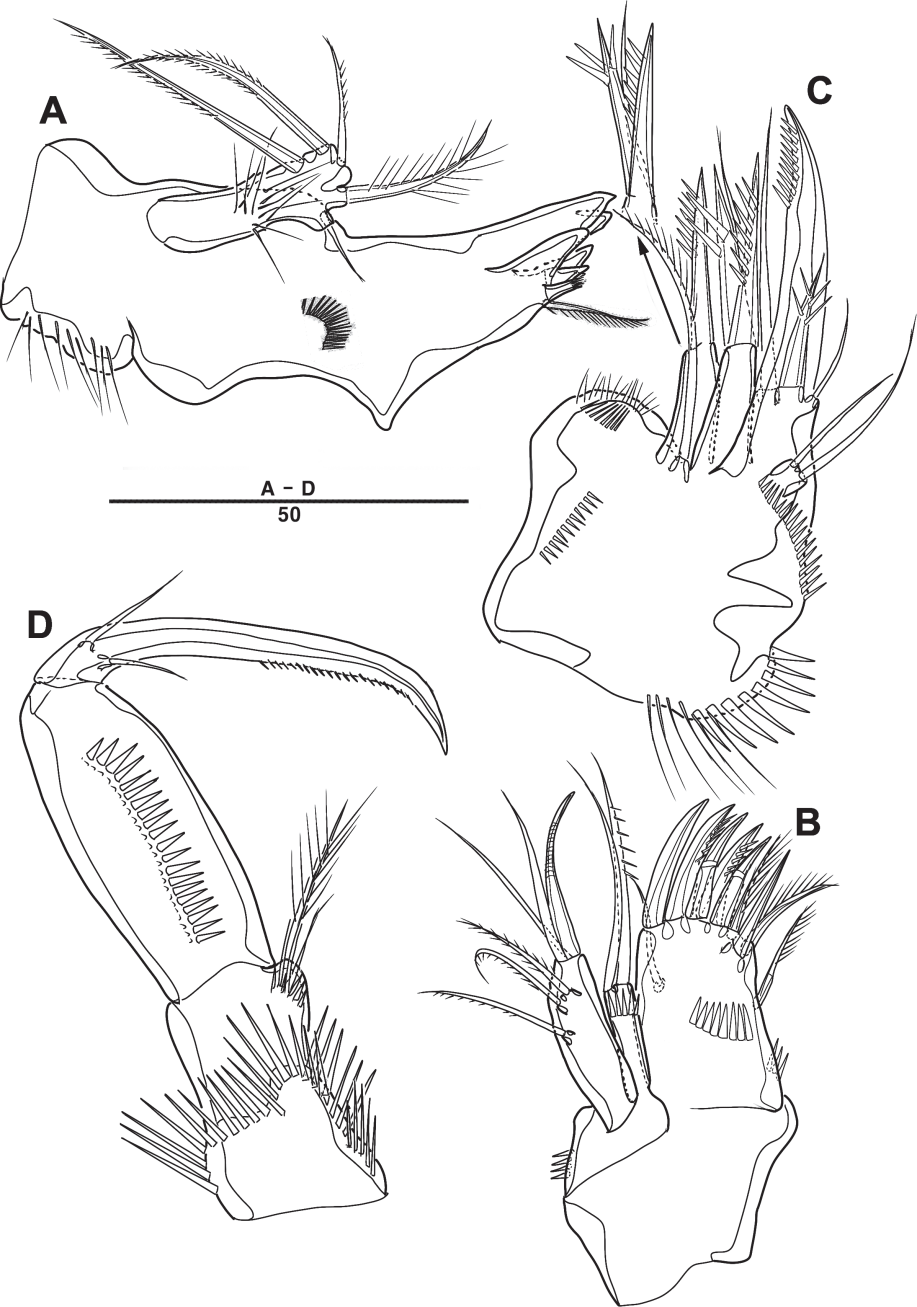
*Orthopsyllus ulsanus* sp. nov., ♀, MABIK CR00259122. **A.**Md; **B.**Mxl; **C.**Mxa; **D.**Mxp. Scale bars in μm.

Md (Fig. 30A): gnathobase with one lateral seta and several blunt teeth, palp one-segmented with five setae, enp, and exp vestigial.

Mxl (Fig. 30B): praecoxa trapezoidal shape; arthrite well developed with one juxtaposed bare seta on anterior surface, one uni-pinnate seta laterally, and eight elements among distal margin; coxal endite with two setae; basis with three setae; enp incorporated in basis, represented by small protrusion with two pinnate setae; and exp represented by one seta.

Mxa (Fig. 30C): syncoxa with three slender endites; proximal endite incorporated into base, represented by one pinnate seta; second and third endites with two spines and one seta; allobasis produced into a strongly curved claw, accessory armature consisting of two bare setae and one spine; and enp represented by two bare setae (Fig. 36C).

Mxp (Fig. 30D): three-segmented; syncoxa with two setae; basis elongate, bare; enp represented by recurved claw with two accessory bare setae near base (Fig. 36B).

**Figure 31.**
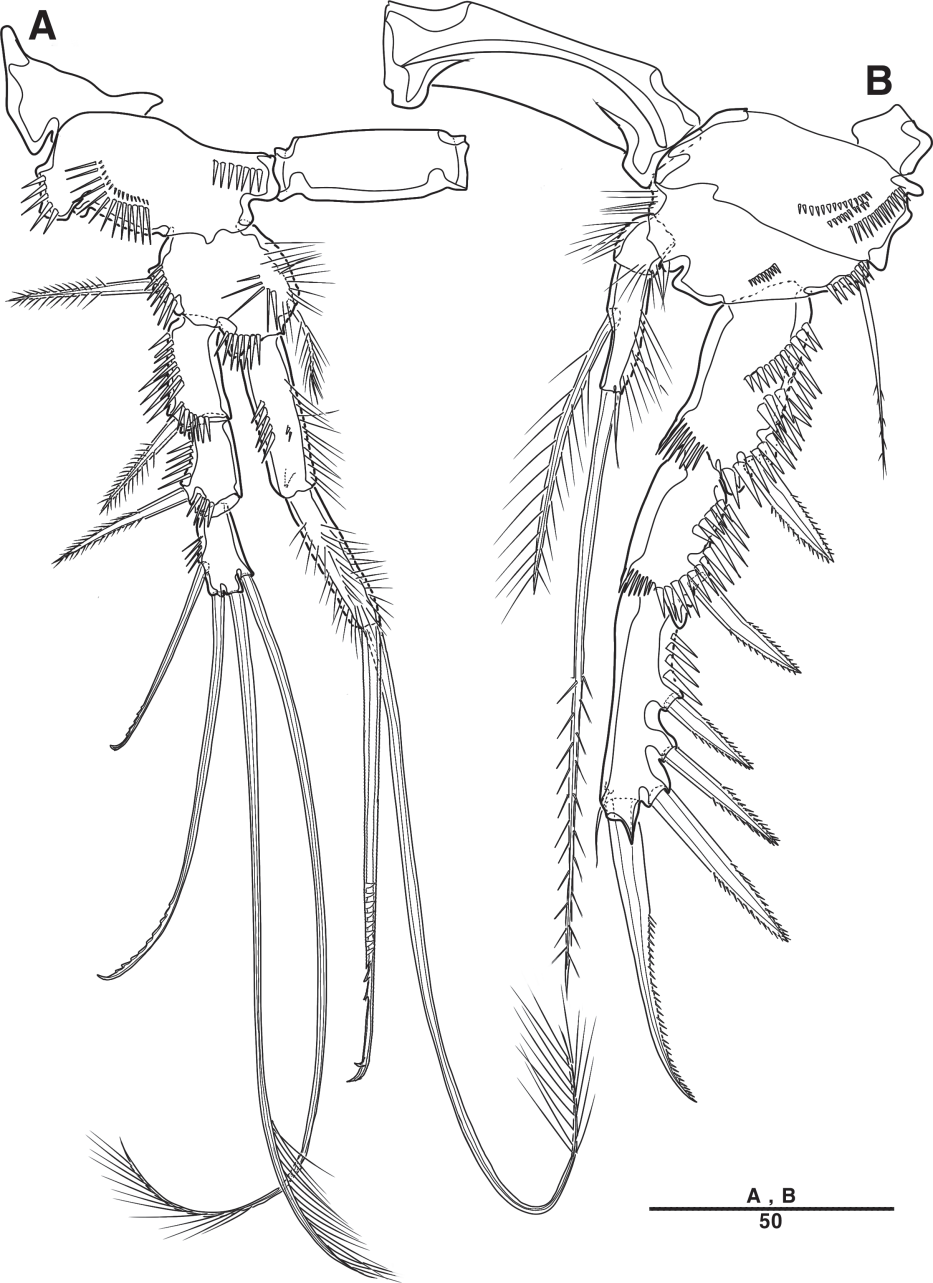
*Orthopsyllus ulsanus* sp. nov., ♀, MABIK CR00259122. **A.** P1; **B.** P2. Scale bars in μm.

P1 (Fig. 31A): coxa bare; basis with one outer and one inner pinnate seta; exp three-segmented; exp–1 and exp–2 each with one outer spine; exp–3 with two outer setae and two brush setae; enp two-segmented; enp–1 bare, ornamented row of long setules among inner margin; and enp–2, with one seta and one brush seta (Fig. 36D).

P2 (Fig. 31B): coxa bare; basis with one outer seta; exp three-segmented; exp–1 and exp–2, each with one outer spine; exp–3 with four spines and one minute seta; enp two-segmented; enp–1, short, bare; enp–2 with one inner pinnate, one apical longest, and one inner shortest seta.

**Figure 32.**
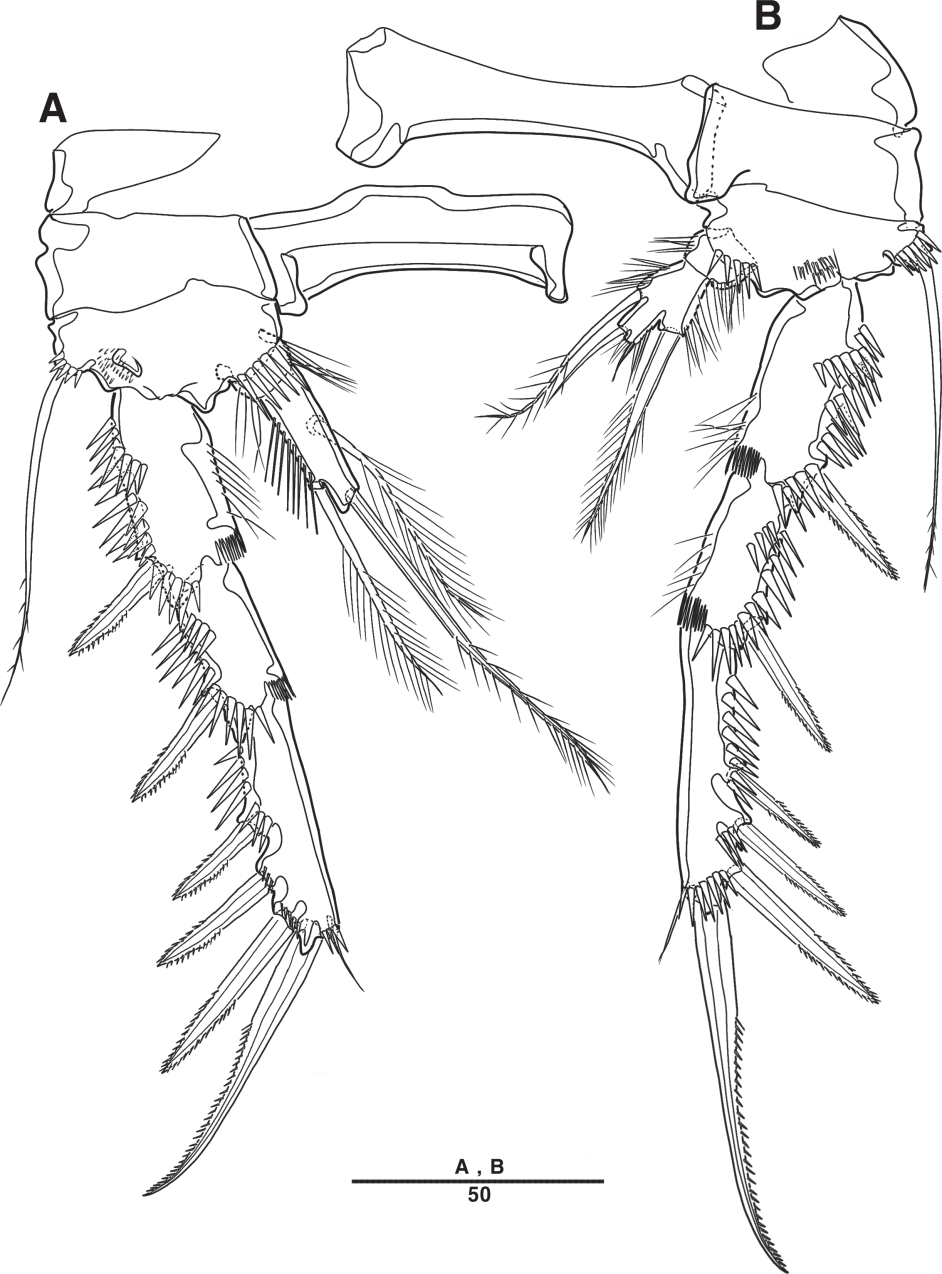
*Orthopsyllus ulsanus* sp. nov., ♀, MABIK CR00259122. **A.** P3; **B.** P4. Scale bars in μm.

P3 (Fig. 32A): coxa bare; basis with one outer seta; exp three-segmented (Fig. 36E); exp–1 and exp–2, each with one outer spine; exp–3 with four spines and one minute seta; enp two-segmented; enp–1, short, bare; and enp–2 with three setae.

P4 (Fig. 32B): coxa bare, basis with one outer seta; exp three-segmented; exp–1 and exp–2 each with one outer spine; exp–3 with four spines and one minute seta; enp two-segmented; enp–1, short, bare; enp–2 with one inner pinnate, two apical setae, and outer strong pinnate seta.

Armature formulae as follows:

**Table:** 

	Exp	Enp
P2	0.0.023	0.111
P3	0.0.023	0.111
P4	0.0.023	0.121

P5 (Fig. 29A): basal part with one outer bare seta; endopodal lobes not fused medially, connected by an intercoxal sclerite, with five pinnate setae; exp defined at base, with six pinnate setae.

P6 (Fig. 29B): P6 vestigial, represented by a small bump bearing two setae.

**Figure 33.**
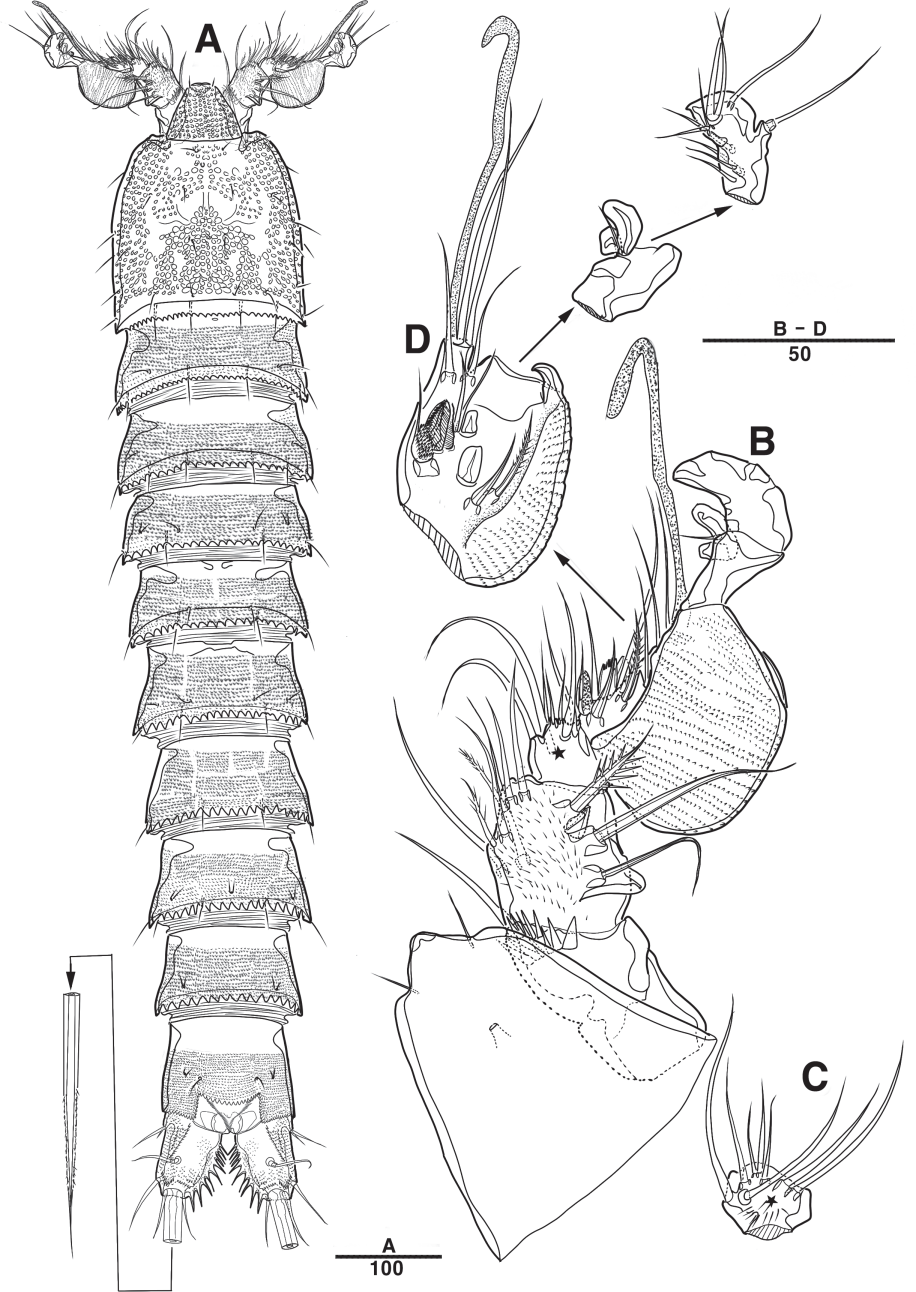
*Orthopsyllus ulsanus* sp. nov., ♂, MABIK CR00259121. **A.** Habitus, dorsal; **B.**A1; **C.** Seg–3 of A1; **D.** Seg–4, –5, and –6 of A1. Scale bars in μm.

Male. Total body (Fig. 33A): length from anterior margin of rostrum to posterior margin of caudal rami 1,000 μm, maximum width 165 μm measured at end of cephalothorax. General body shape, ornamentation, and sensilla pattern as in female except for genital double somite; sexual dimorphism in A1, P3, P4, P5, and P6.

A1 (Fig. 33B–D): six segmented, robust, subchirocer; seg–2 with small blunt process in middle; seg–4 swollen, with two pad-like processes near anterior margin and two blunt processes: 1–[1], 2–[10], 3–[8], 4–[12 + (1 + ae)], 5–[1], 6–[10], apical ae absent, but two apical setae fused at base.

P2 (Fig. 35A): similar to the one of female, except for one outer seta on basis shorter than females and innermost seta on enp–2 longer than females.

P3 (Fig. 35B): exp same as female; enp developed, three-segmented; enp–1 short, bare; enp–2 with long apophysis that apparently tri-segmented apically; and enp–3 with one bare and two pinnate setae.

P4 (Fig. 35C): similar to the one of female, except for enp–2 with one apical (more robust than female), one bare, and two pinnate setae.

**Figure 34.**
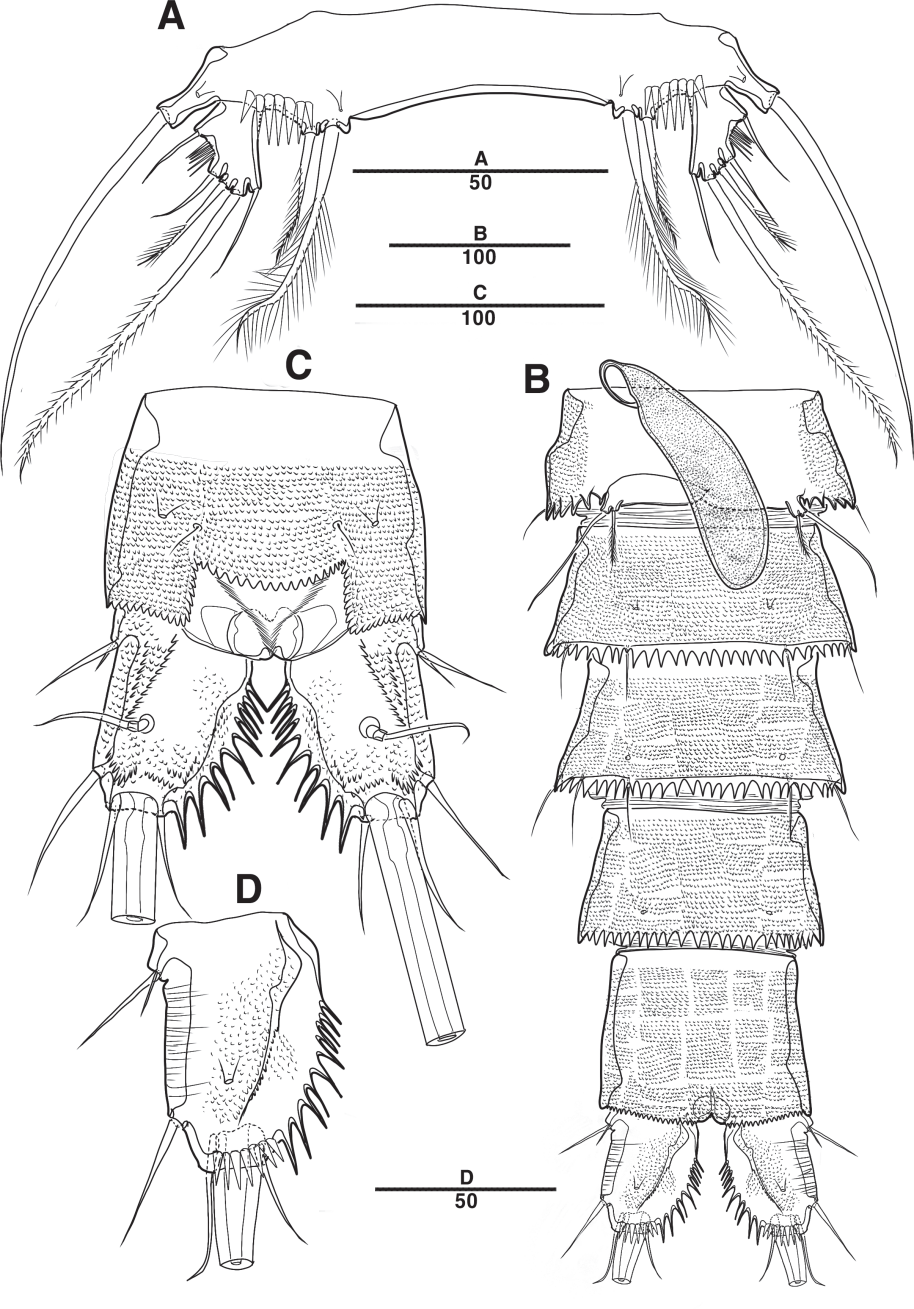
*Orthopsyllus ulsanus* sp. nov., ♂, MABIK CR00259121. **A.** P5; **B.** Urosome, ventral; **C.** Anal somite and caudal rami, dorsal; **D.** caudal rami ventral. Scale bars in μm.

P5 (Fig. 34A): benp represented by a small protrusion with two pinnate setae; exp defined at base with five setae.

**Figure 35.**
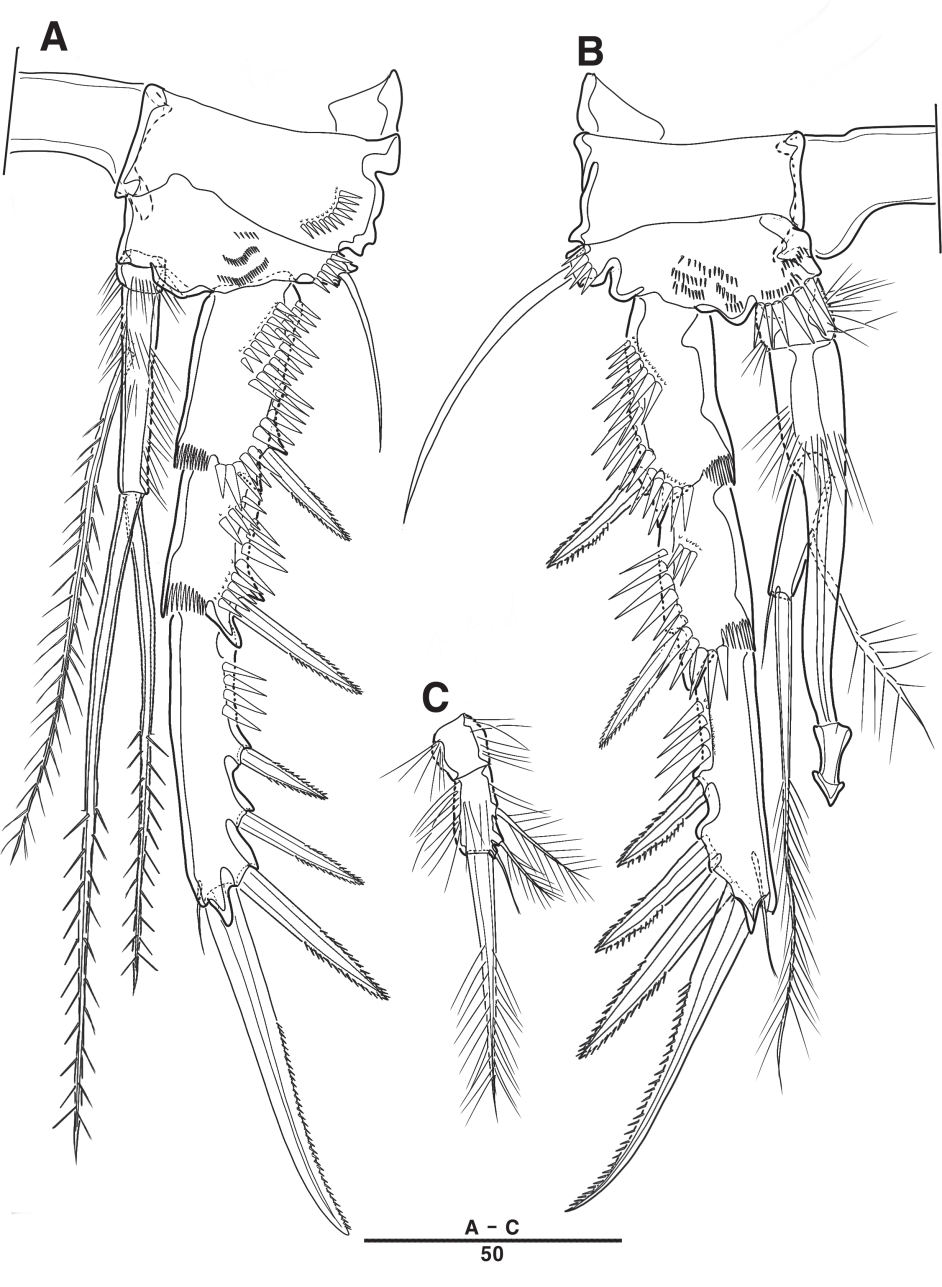
*Orthopsyllus ulsanus* sp. nov., ♂, MABIK CR00259121. **A.** P2; **B.** P3; **C.** P4 enp. Scale bars in μm.

**Figure 36.**
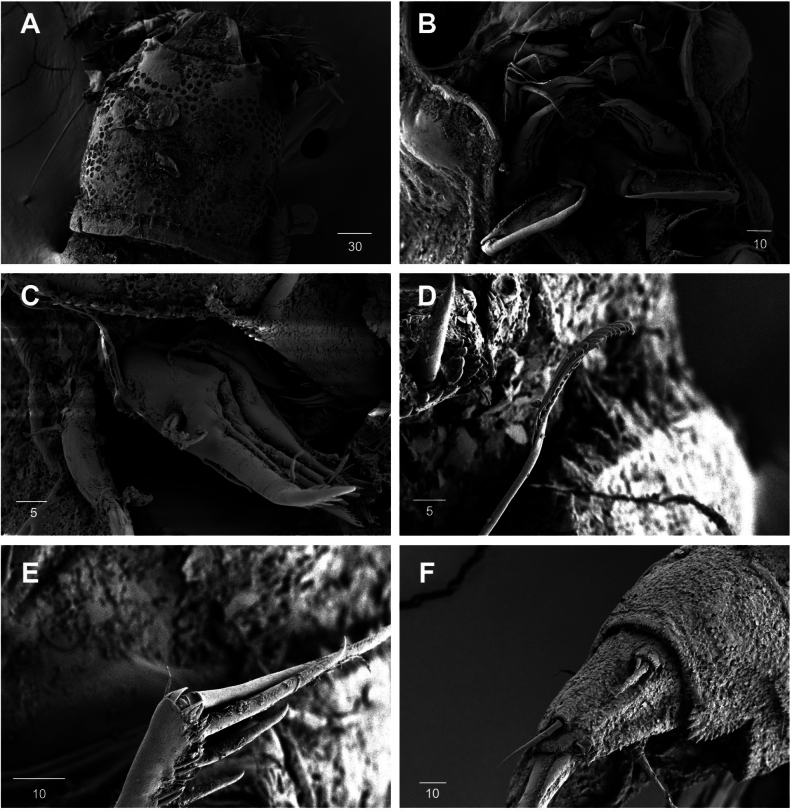
*Orthopsyllus ulsanus* sp. nov., ♀, SEM, **A.** Rostrum and cephalothorax; **B.** Mouth part; **C.**Mxa; **D.** Brush seta on P1 enp–2; **E.** P3 exp–3; **F.** Caudal rami. Scale bars in μm.

P6 (Fig. 34B): asymmetrical, with functional right member articulating at base and closing off genital aperture, and left member fused at base to genital somite; each P6 with one bare and one pinnate seta.

Caudal rami (Fig. 34C, D): general shape same as in female; inner lamella ornamented with ten teeth.

##### Etymology.

The species name refers to the type locality.

### ﻿Molecular results

**Figure 37.**
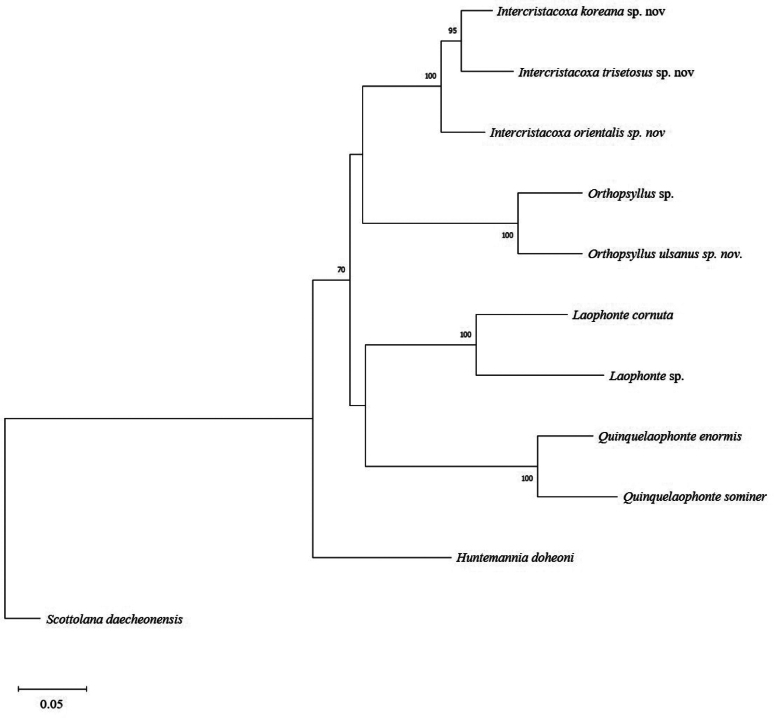
Maximum likelihood phylogenetic tree reconstructed from concatenated mtCOI and 18S rRNA sequences, showing relationships among four genera of the superfamily Laophontoidea, with the family Canuellidae as the outgroup. Numbers on branches indicate bootstrap support values (ML, %). The scale bar indicates branch lengths measured in the number of substitutions per site.

The intraspecific pairwise distance for the mtCOI gene was highest in *Intercristacoxa orientalis* sp. nov., with a value of 4.262%. For the 18S rRNA gene, all three new species of *Intercristacoxa* gen. nov. showed identical sequences, while *Orthopsyllus ulsanus* sp. nov. exhibited a maximum intraspecific difference of 0.168% (Table 1). Based on these results, a Maximum Likelihood (ML) tree was constructed using concatenated mtCOI (657 bp) and 18S rRNA (1,700 bp) sequences (Fig. 37).

## ﻿Discussion

### ﻿Taxonomic position of the new genus *Intercristacoxa* gen. nov.

The new genus can be placed in the family Orthopsyllidae, based on the combination of the following characters: 1) the shape of the mandibular palp; 2) the exp of antenna is 1-segmented; 3) the distal segment of the P1 exp is armed with two long apical setae; 4) the well-developed endopodal lobe of the female P5 is not medially fused but connected by an intercoxal sclerite; 5) the female P5 benp and exp are distinct; and 6) an apophysis appears on the second segment of the male P3.

The most prominent distinguishing feature of the new genus is the four-segmented A1 and the presence of a thorn-like process on the outer margin of the first antennular segment. The primitive form of the A1 in the superfamily Laophontoidea is thought to be eight-segmented. The four-segmented A1 appears as a fusion of the third and fourth segments and the fifth to eighth segments in the A1 of Orthopsyllidae (Huys 1990). The processes that were present on the first and second segments of the primitive species remain only on the second segment in *Orthopsyllus* and the first segment in *Intercristacoxa* gen. nov. However, Huys (1990) mentioned that the difference in the location of the process is not a very important character. In addition, the ae appears only in the third segment in Orthopsyllidae and the apical ae degenerates during the fusion process from segments V to VIII, leaving traces of a form in which two or three bases are fused without an ae.

**Table 1.** GenBank accession numbers and intraspecific pairwise distances for the mtCOI and 18S rRNA gene sequences of new and related species. N/A = Not applicable.

	Accession number		Intraspecific pairwise distance % (Min. – Max.)	
	mtCOI	18S rRNA	mtCOI	18S rRNA
Family Orthopsyllidae				
Genus *Intercristacoxa* gen. nov.				
*I. orientalis* sp. nov.	PX210751, PX210752, PX485055 – PX485058	PX218728, PX218729	0.152–4.262 (*n* = 6)	0 (*n* = 2)
*I. koreana* sp. nov.	PX210748 – PX210750, PX485050 – PX485054	PX218726, PX218727, PX485043 – PX485045	0–0.462 (n = 8)	0 (n = 5)
*I. trisetosa* sp. nov	PX210753	PX218730, PX218731	N/A	0 (*n* = 2)
Genus *Orthopsyllus*				
*O. ulsanus*	PX210754, PX210755, PX485059 – PX485064	PX218732, PX218733, PX485046 – PX485049	0–1.976 (*n* = 8)	0–0.168 (*n* = 6)
*Orthopsyllus* sp.	PX608304 – PX608309	PX606910, PX606911	0.152–1.065 (*n* = 6)	0.042 (*n* = 2)
Family Laophontidae				
Genus *Laophonte*				
*L. cornuta*	PV110166,	PV112071,	N/A	N/A
*Laophonte* sp.	PV110177	PV112076	N/A	N/A
Genus *Quinquelaophonte*				
*Q. enormis*	MT416598 – MT416603, PV189950	MT410708, PV189457	0–1.199 (*n* = 7)	0.167 (*n* = 2)
*Q. sominer*	OR659900 – OR659928	OR656936 – OR65938	0–1.220 (*n* = 29)	0 (*n* = 3)
Family Nannopodidae				
Genus *Huntemannia*				
*H. doheoni*	PX608300 – PX608303	PX606908, PX606909	0.304–0.913 (*n* = 4)	0 (*n* = 2)
Family Canuellidae				
Genus *Scottolana*				
*S. daecheonensis*	OP452900	OP454041	N/A	N/A

The second important characteristic of the new genus is the morphology of P1. The known P1 characters in *Orthopsyllus* are as follows: 1) absence of an inner seta on enp–1; 2) presence of one brush seta and one long, thin seta with modified claws on enp–2; 3) absence of an inner seta on exp; and 4) absence of an outer spine in the distal segment of exp. Particularly, P1 enp–2 of the new genus is armed with a strong claw and a distally pectinate seta. Huys (1990) suggested that the ancestors of Laophontoidea probably had two geniculate setae on the distal segment, which are thought to have undergone several alterations in different lineages. The setae on the second segment of the P1 in the new genus are considered to have evolved through heterochrony, adapting to prey capture and feeding in a burrowing, sedimentary environment.

The third notable characteristic is the sexual dimorphism in the male P3 enp. The female P3 enp is one-segmented, whereas the male P3 enp is two-segmented in the new genus. This suggests that the first segment of the P3 enp was once present but has since degenerated, as in the case of *Orthopsyllus* (Huys 1990). Also, the P3 enp of the adult male in *Orthopsyllus* is three segments; however, in the new genus, the second and third segments are incompletely fused. The characters of the apophysis are very similar to the homologous structures of the apophysis of *Orthopsyllus* proposed by Huys (1990), and the morphology of the P3 segment of male in three new species of *Intercristacoxa* gen. nov.

The morphological features of the new genus (coxal crests on the P1, a claw-like spine instead of two brush setae on the P1 enp–2, and a thorn-like process on the first rather than the second segment of A1) do not appear to represent homologous characters justifying its placement within Cristacoxidae. These traits are instead interpreted as a result of heterochrony, arising from adaptation to the habitat of *Intercristacoxa* gen. nov. As noted by Boxshall and Huys (1992), a reduction in the number of segments and simplification of appendages is a common occurrence in harpacticoids inhabiting sediment surfaces or burrows. The development of coxal process in burrowing copepods is documented in the nannopodid genera *Huntemannia*, *Rosacletodes*, *Acuticoxa*, and *Laophontisochra*. Furthermore, the crest on the P1 coxa, similar to that seen in both the Cristacoxidae and *Intercristacoxa* gen. nov., has been reported in *Concilicoxa hispida* Kim & Lee, 2020 from sandy sediments (Kim and Lee 2020). This suggests that the burrowing behavior of the new genus, found in the sediment of broken rock crevices, drove the convergent evolution of these features, including the P1 coxal crests, the loss of the P1 enp brush seta, and the degeneration of the P2–P4 enp and exp. Conversely, the new genus shares homologous characters with Orthopsyllidae, such as the morphology of the female P5 and the male P3 apophysis. These characters are interpreted as underpinning a strategy involving neoteny and clasping, consistent with the observed habitats and adaptations.

Huys and Kihara (2010) proposed fourteen autapomorphies for the family Cristacoxidae, including twelve previously discussed by Huys (1990) and two new ones. The inclusion of *Intercristacoxa* gen. nov. within Cristacoxidae is rendered difficult by its incongruence with the following six of fourteen autapomorphic characters: 1) The second endite of the Mxa bears one modified, basally fused spine (this endite and spine are distinct in the new genus); 2) a serrate crista is present on the P1 praecoxa (this crista is absent in the new genus), with homologous crests also occurring on the P2 and P3 coxae (crest on P2 and P3 coxae is absent in the new genus); 3) the P1 exp–3 possesses four geniculate setae (the new genus has two long setae and three short setae); 4) the P2–P4 exp–2 lacks an inner seta (some species of the new genus have one inner seta); 5) the P5 is paedomorphic, forming a common plate in both sexes (In the new genus, the P5 of the female consists of a well-developed benp connected by an intercoxal sclerite, while in both sexes the exp is separated from its base.). The following characters: 1) The thorn-shaped structure on the first segment of A1; 2) the first endite of the Mxa represented by one seta; 3) the presence of serrated cristae in the P1 coxa; 4) the 1-segmented P3 and P4 enp; and 5) a bi-articulated form of the caudal seta V is considered a result of convergent evolution because they are similar to those of the *Intercristacoxa* gen. nov. and Cristacoxidae. Furthermore, following additional characters make it clear to include the new genus in Orthopsyllidae: 1) The A2exp is 1-segmented; 2) the P5 has a developed benp and a separate exp armed with six setae; 3) the shape of the mandibular palp; 4) the caudal rami without spinous processes at the inner distal corner; and 5) the presence of an apophysis and one or more inner setae in male P3 enp–2.

### ﻿Diagnosis update of family Orthopsyllidae

After Huys (1990) discussed the concept and morphological characters of the superfamily Laophontoidea, the phylogenetic discussion on the morphological characters appearing at the family level has continued. In particular, the 59 morphological characters proposed by Gómez and Nazari (2021) for phylogenetic analysis of the superfamily Laophontoidea are very useful for the diagnosis of Orthopsyllidae. Based on the previous phylogenetic analyses of the relationships among families in Laophontoidea (Huys 1990; Huys and Lee 1998; Huys and Kihara 2010; Gómez and Nazari 2021), the diagnosis of Orthopsyllidae, including the new genus, is updated and presented as follows:

Laophontoidea. Female. Habitus cylindrical, without clear demarcation between prosome and urosome. Rostrum well-developed, defined at base and triangular. Cephalothorax with cuticular pits in middle. Genital double-somite separated dorso-laterally but completely fused ventrally. Anal operculum semicircular. Caudal rami semi-conical. A1 four-segmented in females, with one spinous process on seg–1 and/or seg–2, apical ae not present. A2exp uni-segmented, with 2–4 setae. Mdexp incorporated into base, with one seta. Mxlenp incorporated into allobasis. Mxp prehensile; syncoxa with one or two seta(e), basis without seta; enp one-segmented, represented by one long claw with one or two accessory seta(e). P1 exp one- – three-segmented, exp–1 and exp–2 without inner seta, distal segment with four or five setae (without spine); enp bi-segmented, enp–1 without inner seta, and enp–2 with at least one modified seta (modified means brush, geniculate, saw-like, pectinate, claw, or slender claw). P2–P4, distal exp segment at least two spines; proximal enp segment reduced. P5 endopodal lobe not fused medially, connected by intercoxal sclerite; exp not fused at base; endopodal lobes with four or five setae, exp with six setae. P6 represented by one or two seta(e).

### ﻿Sexual dimorphism in genital somite, A1, P2, P3 enp, P5, and P6

Male. A1 subchirocerous, six or seven segments. P3 enp two or three-segments; seg–2 with one outer apophysis. P5 exp not fused at base. P6 asymmetrical, only one leg functional, with two setae.

### ﻿Key to genera and species in the Orthopsyllidae

(This key is based on the female characters, except for number 9, 13, 14, and 21.)

The three new species in *Intercristacoxa* gen. nov. share the reduced appendages in their mouthparts and swimming legs compared to those in *Orthopsyllus*.

The primary morphological difference among the three new species in the new genus is the number of setae on the P3 and P4 enp. Differences also exist in the number of setae on the P5 endopodal lobe. Furthermore, all three new species exhibited an abnormality in which the seta lengths of the left and right P5 endopodal lobes were different. Sexual dimorphism occurs in the male P4 enp, with all females having three elements, regardless of the number of setae.

Some species of the genus *Orthopsyllus* require taxonomic revision due to missing characters, misrepresentation, or difficulties in distinguishing between or within species in previous studies (Boer 1971; Huys 1990; Bodin 1997; Wells 2007). Boer (1971) mentioned ten reasons for the difficulty in the classification of the *Orthopsyllus*, including the lack of clear descriptions of the characters of the species (*O. linearis*, *O. spinicaudatus*, *O. improportionatus*, *O. wallini*, *O. major*, *O. agnatus*, and *O. propinquus*). He also discussed intraspecific variation and organized the existing species into four groups (*O. linearis*, *O. wallini*, *O. sarsi*, and *O. spinicaudatus*). With regard to intraspecific variation or subspecies, such as differences in the shape of the caudal lamella and caudal seta V in the seven specimens of *O. illgi* illustrated by Lang (1965), further research is needed on intraspecific variation and species-level differences (Suppl. material 1). Obtaining additional genetic information from several species would be helpful. Because it is difficult to reflect all variations represented by a single species, the key is based on the annotated papers.

Apart from a single record of Orthopsyllus cf. linearis, no other species of *Orthopsyllus* from Korean waters has been described to the species level (MBRIS 2015). However, Park et al. (2012) failed to designate the species due to insufficient morphological information from the original description of *O. linearis linearis* (Claus, 1866) and the difficulty in comparing its characteristics with those of its subspecies (*O. linearis bulbosus* Noodt, 1955, *O. linearis curvaspina* Mielke, 1993; *O. linearis setosus* Boer, 1971). The newly discovered species, *O. ulsanus* sp. nov., is differentiated from other known species of *Orthopsyllus* by the following combination of characters: 1) A well-developed, serrate lamella on the inner margin of the caudal rami; 2) four setae on the P4 enp; 3) the absence of an inner seta on the P2 exp–2; 4) three setae on the P3 enp–3 in males; 5) a normally shaped caudal seta V, lacking a swollen base. To accurately differentiate species within *Orthopsyllus*, further research should focus on a more detailed examination of specific characters, including the number and morphology of setae on the male P5 and P6, the number of setae on the Mxp coxa, and the shape of caudal seta V.

Although partial gene sequences may provide limited information for constructing accurate phylogenetic tree (Bernot et al. 2025), concatenated mtCOI and 18S rRNA sequence data provide valuable information for both species and genus-level classification (Fig. 37). The taxonomic position of the new genus, within the family Orthopsyllidae was not statistically significant, as indicated by very low bootstrap values (< 50%) in the phylogenetic trees based on mtCOI and 18S rRNA data (Fig. 37). Additional genetic information from Cristacoxidae, Laophontopsidae, and Adenopleurellidae will be necessary to enhance our understanding of the phylogenetic relationships among the taxa within the superfamily Laophontoidea.

**Table:** 

1	Thorn-like (outgrowth) process in A1seg–1; P1 enp–2 with one thorn-like seta/spine; P1 coxa with outer serrate crests (*Intercristacoxa* gen. nov.)	**2**
–	Thorn-like (outgrowth) process in A1seg–2; P1 enp–2 and exp–3 with brush seta; P1 coxa normal (*Orthopsyllus* Brady & Robertson, 1873)	**4**
2	P3 enp with 2 setae	***I. koreana* sp. nov.**
–	P3 with 3 setae	**4**
3	Benp with 4 setae; P4 enp with 3 setae	***I. trisetosa* sp. nov.**
–	Benp with 5 setae; P4 enp with 2 setae	***I. orientalis* sp. nov.**
4	P2 enp 1-segmented	**5**
–	P2 enp 2-segmented	**6**
5	P2 enp with 2 seta	***O. improportionatus* (Jakobi, 1954)** ^1^
–	P2 enp with 1 setae	***O. spinicaudatus* Krishnaswamy, 1957** ^2^
6	P1 enp–1 with 1 inner seta	***O. sarsi* Klie, 1941** ^3^
–	P1 enp–1 without inner seta	**7**
7	P4 enp–1 with 1 inner seta	***O. rugosus* Nicholls, 1941** ^4^
–	P4 enp–1 without inner seta	**8**
8	P2 exp–2 with 1 inner seta	***O. koprii* Lee, Gheerardyn & Lee, 2011** ^5^
–	P2 exp–2 without inner seta	**9**
9	Male P3 enp–3 (distal segment) with 1 seta	**10**
–	Male P3 enp–3 (distal segment) with at least 2 setae	**11**
10	P4 enp–2 with 1 seta; P1 basis without inner seta	***O. littoralis* Nicholls, 1942** ^6^
–	P4 enp–2 with 3 setae; P1 basis with 1 inner seta	***O. wallini* Lang 1934** ^7^
11	Inner lamella of caudal rami developed, serrated or ornamented with a row of strong spinules	**12**
–	Inner margin of caudal rami smooth or ornamented with small spinules	**15**
12	P2 and P3 enp–2 with 2 setae	***O. similis* Nicholls, 1942** ^6^
–	P2 with 3 setae at least	**13**
13	Male P6 with 2 setae; male P3 enp–3 with 3 setae; male P4 enp–2 with 4 setae	***O. ulsanus* sp. nov.**
–	Male P4 enp–2 with 3 setae; these characters not combined	**14**
14	Male P6 with 1 seta; male P3 enp–3 with 3 setae	***O. dubius* Vervoort, 1964** ^8^
–	Male P6 with 2 setae; male P3 enp–3 with 2 setae	***O. pectinicauda* Vervoort, 1964** ^8^
15	P1 enp–1 approximately twice as long as enp–2	**16**
–	P1 enp segments equal in length	**17**
16	P2 enp–2 with 2 setae	***O. coralliophilus* Fiers, 1987** ^9^
–	P2 enp–2 with 3 setae	***O. major* Klie, 1939** ^10, 11^
17	P5 exp with 5 setae	***O. illgi* (Chappuis, 1958)** ^12^
–	P5 exp with 6 setae	**18**
18	Basal part of caudal seta V not bulbous	**19**
–	Basal part of caudal seta V bulbous	**20**
19	P2 enp–2 with 1 seta	***O. linearis linearis* (Claus, 1866)** ^3^
–	P2 enp–2 with 3 setae	***O. agnatus* Klie, 1950** ^13^
20	P2 enp–2 with 2 setae	***O* . *linearis setosus* Boer, 1971** ^14^
–	P2 enp–2 with 3 setae	**21**
21	Male P2 enp–1 not reduced	***O. linearis bulbosus* Noodt, 1955** ^15^
–	Male P2 enp–1 reduced; innermost spine of male P3 exp–3 strongly bent inwardly	***O. linearis curvaspina* Mielke, 1993** ^16^

^1^Jakobi, 1954; ^2^Krishnaswamy, 1957; ^3^Lang, 1948; ^4^Nicholls, 1941; ^5^Lee, Gheerardyn & Lee, 2011; ^6^Nicholls, 1942; ^7^Lang, 1934; ^8^Vervoort, 1964; ^9^Fiers, 1987; ^10^Klie, 1939; ^11^Klie, 1941; ^12^Chappuis, 1958; ^13^Klie, 1950; ^14^Boer, 1971; ^15^Noodt, 1955; ^16^Mielke, 1993.

## Supplementary Material

XML Treatment for Intercristacoxa

XML Treatment for Intercristacoxa orientalis

XML Treatment for Intercristacoxa koreana

XML Treatment for Intercristacoxa trisetosa

XML Treatment for Orthopsyllus ulsanus

## ﻿Additional information

### Use of AI

No use of AI was reported.

### Author ORCIDs

Jinwook Back https://orcid.org/0000-0003-0285-1135

Hyun Woo Bang https://orcid.org/0000-0002-9626-9762

### Data availability

All of the data that support the findings of this study are available in the main text or Supplementary Information.

## Supplementary materials

Supplementary material 1 Main characters used for species identification of the family Orthopsyllidae Data type xlsx https://binary.pensoft.net/file/1508622This dataset is made available under the Open Database License (http://opendatacommons.org/licenses/odbl/1.0/). The Open Database License (ODbL) is a license agreement intended to allow users to freely share, modify, and use this Dataset while maintaining this same freedom for others, provided that the original source and author(s) are credited.Jinwook Back, Hyun Woo Bang
